# Atomic Structure and 3D Shape of a Multibranched Plasmonic Nanostar from a Single Spatially Resolved Electron Diffraction Map

**DOI:** 10.1021/acsnano.4c05201

**Published:** 2024-09-21

**Authors:** Leonardo M. Corrêa, Simon M. Fairclough, Kaleigh M. R. Scher, Supriya Atta, Diego Pereira dos Santos, Caterina Ducati, Laura Fabris, Daniel Ugarte

**Affiliations:** † Instituto de Física Gleb Wataghin, Universidade Estadual de Campinas, Campinas 13083-859, Brazil; ‡ Department of Materials Science and Metallurgy, University of Cambridge, Cambridge CB3 0FS, U.K.; § Department of Materials Science and Engineering, Rutgers University, Piscataway, New Jersey 08854, United States; ∥ Department of Biomedical Engineering, Duke University, Durham, North Carolina 27708, United States; ⊥ Instituto de Química, Universidade Estadual de Campinas, Campinas 13083-859, Brazil; # Department of Applied Science and Technology, Politecnico di Torino, Turin 10129, Italy

**Keywords:** plasmonic nanoparticles, nanostar, 4D-STEM, precession electron diffraction, crystal orientation mapping, nanoparticle morphology, nanocrystallography

## Abstract

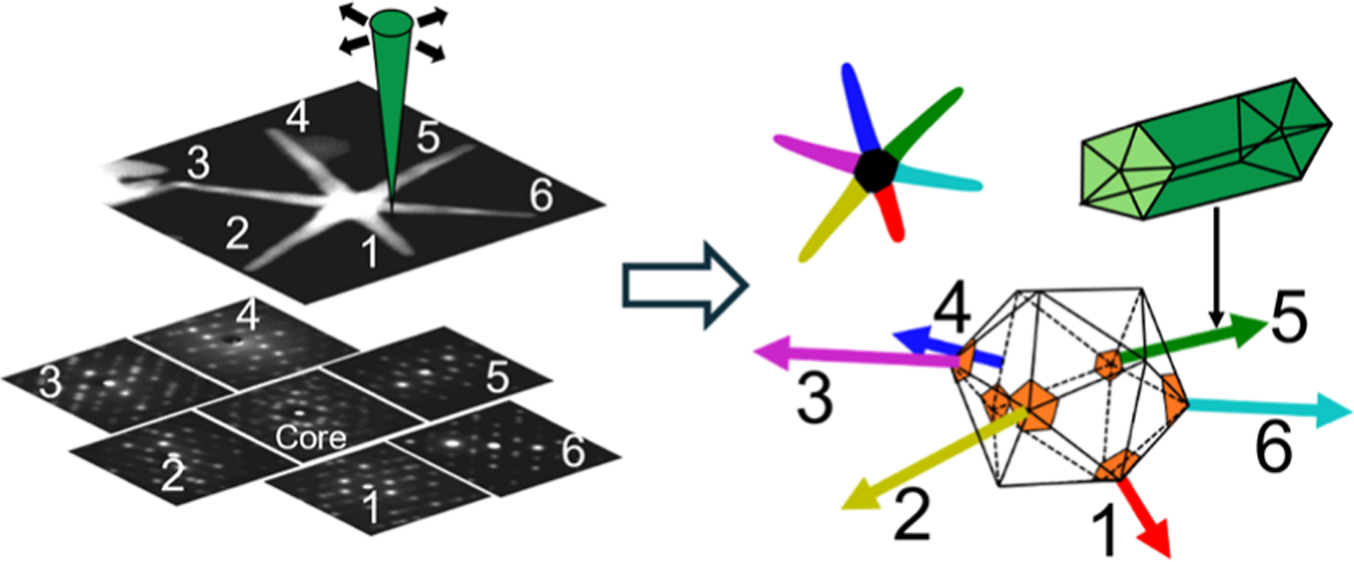

Despite the interest in improving the sensitivity of optical sensors using plasmonic nanoparticles (NPs) (rods, wires, and stars), the full structural characterization of complex shape nanostructures is challenging. Here, we derive from a single scanning transmission electron microscope diffraction map (4D-STEM) a detailed determination of both the 3D shape and atomic arrangement of an individual 6-branched AuAg nanostar (NS) with high-aspect-ratio legs. The NS core displays an icosahedral structure, and legs are decahedral rods attached along the 5-fold axes at the core apexes. The NS legs show an anomalous anisotropic spatial distribution (all close to a plane) due to an interplay between the icosahedral symmetry and the unzipping of the surfactant layer on the core. The results significantly improve our understanding of the star growth mechanism. This low dose diffraction mapping is promising for the atomic structure study of individual multidomain, multibranched, or multiphase NPs, even when constituted of beam-sensitive materials.

Noble metal nanoparticles (NPs) attract huge interest due to the possibility of tuning their optical properties. This usually exploits the excitation of surface plasmon resonances, which show a strong dependence on dielectric properties, morphology, size, and interparticle distance.^1^ Optical metamaterials^2^ and surface-enhanced Raman spectroscopy represent actual application of tuned optical response, where efficiency is deeply tied to NP morphologies. Despite its relevance, the determination of three-dimensional (3D) shapes of very complex NPs (anisotropic, branched, etc.) is an everlasting issue in nanoscience.^1^ Many different morphologies of metal NPs have been synthesized and proposed to generate strong plasmonic response, such as nanospheres, nanorods, nanosheets, and nanostars (NSs).^3^ Among them, multibranched plasmonic NSs display high field enhancement around the sharp tips of high-aspect-ratio legs.^1,4^ The precise understanding of the NS structure and 3D shape is an essential step to refine synthesis protocols, to help tailor shape, and to optimize plasmonic response.

Transmission electron microscopy (TEM) and its related scanning mode (STEM) represent the best suited tools to characterize nanomaterials due to their intrinsic high spatial resolution.^5−7^ TEM (or STEM) images just provide 2D projections of the studied object, and the 3D structure can be reconstructed by applying discrete electron tomography (ET) to a series of 50–70 images taken at different tilting angles (usually in a tilt range −70 to 70°).^8^ In the field of nanomaterials, ET may exploit high-angle annular dark-field (HAADF) STEM images to derive particle faceting using nanometer-wide electron probes, and atomic resolution ET reconstructions may be generated exploiting aberration-corrected instruments. This requires the generation of Angstrom-size probes with semiconvergence angles of 25–30 mrad; this convergence limits the depth of field to 5–7 nm (resolution along the longitudinal electron beam path) to avoid image resolution loss.^8−11^ Therefore, atomic structure ET is restricted to particles that are thin (10–20 nm), and images must be acquired at high magnification. This limits its use to rather small regions when a large particle is studied. Despite recent progress,^8−12^ STEM ET remains quite demanding considering the required beam time (typically 1 h acquisition time) and high electron irradiation doses (10^5^–10^6^ e^–^/Å^2^);^8^ thus, beam-sensitive samples can be seriously modified.

New opportunities to study nanosystems have recently been explored by the so-called 4-dimensional scanning-TEM (4D-STEM),^13^ where either an electron diffraction (ED) or a scattering pattern is stored for each image pixel position (see Figure S1). This method allows the measurement of, for example, atomic arrangement, phase, crystal orientation, and strain, with nanometer resolution^13^ using a much lower irradiation dose.^14^ Additionally, a 4D-STEM data set maps local crystallographic information, and it can be used to get the spatial distribution of regions that generate a particular crystal attribute in a reciprocal space (e.g., individual peaks and the related virtual dark-field images: VDF).^13,15^

In this work, we address the study of a complex six-branched AuAg plasmonic NS, where branches show a high aspect ratio (7–10 nm in diameters and 35–60 nm in length); the star structure attains a diameter in the 150–200 nm range. These NSs are synthesized by seed-mediated methods exploiting peculiarities of different surfactants. The deep understanding of the star optical properties and synthesis protocols requires the precise measurement of different structural aspects: (a) 3D spatial distribution of the legs, (b) atomic arrangement inside seeds and legs, and (c) how the seeds structure determines leg number and their spatial configuration. The application of ET tomography to answer all of these questions may be extremely challenging. First, atomic resolution ET cannot be used to analyze such extended objects (50–70 nm in length) because the necessary depth of field would be ∼70 nm. This will limit the convergence angle to about 1.0–1.5 mrad, and then the generated electron probe will be in the nanometer-wide range; ET using this kind of electron beams may be used to measure leg distribution but will be unable to provide atomic arrangement knowledge. Some studies have overcome these difficulties by taking a nanometer-resolution ET to determine 3D morphology and perform a second atomic resolution ET of a very reduced NP region such as the tip of a nanowire or the corner of a NP.^16^ Here, we have adopted a different strategy to determine NS atomic structure with reduced electron irradiation; we show that the crystallographic information mapped by a 4D-STEM data set can provide simultaneous determination of leg 3D distribution, atomic arrangement of legs and seed, and their orientation relationships. All this NS structural information could be derived from one single 4D-STEM diffraction map using a total electron dose of ∼200 e^–^/Å^2^.

## Crystalline Structure of the NS Legs

**Figure 1:**
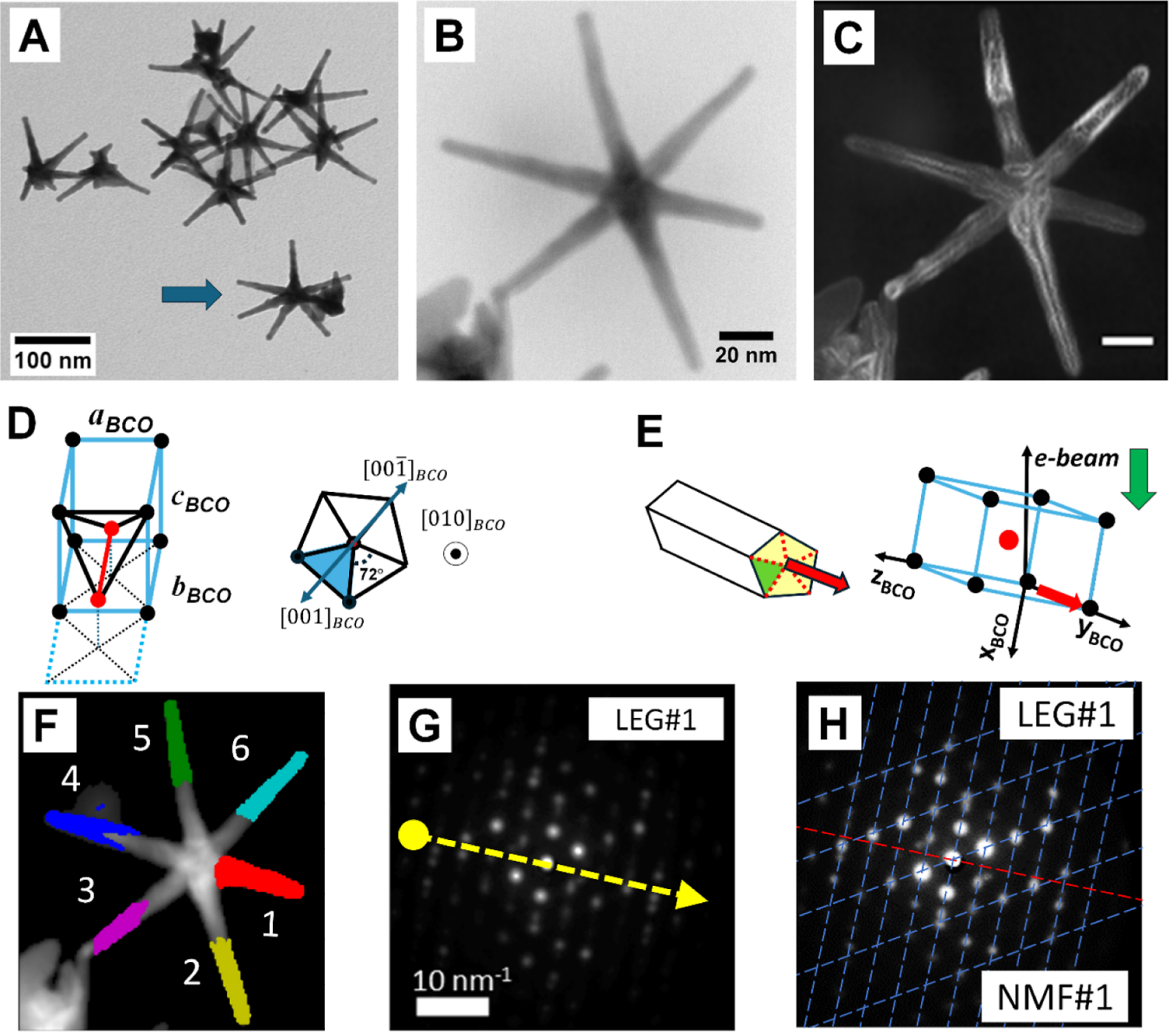
(A) TEM micrograph displaying general view of a high-aspect-ratio AuAg NS. (B) BF-STEM image of the NS studied in detail by 4D-STEM precession diffraction methods. (C) A virtual anticorrelation image revealing abrupt crystallographic changes between pixels (pixel step 1 nm) inside legs, which confirms their decahedral structure. (D) BCO unit cell and the relevant crystallographic directions in relation to a decahedral structure ([010]_BCO_ vector points along the decahedral leg axis as derived in Figure S3). (E) The crystal orientation deduced from the ED pattern interpretation that allows the determination of LEG#1 orientation in space (b_BCO_ vector points downward in the scheme) in relation to the incident electron beam (or in sample coordinates). (F) A virtual annular dark field (VADF) image generated from the 4D-STEM data set. The colored region at legs’ tips mark regions of pixels clustered by the machine learning (ML) tool *K*-means; the regions of all 6 legs have been recognized (indicated numbers are used to identify different legs in the subsequent sections of this work, see the Methods section for details and explanations). (G) A mean diffraction pattern calculated from the region on LEG#1 (the arrow indicates the leg axis on the pattern along the base–tip direction as derived from the annular VDF image). (H) Interpretation of the mean diffraction pattern of LEG#1 in (G) was rendered easier after using non-negative matrix factorization (NMF) to perform a partial demixing of the diffraction contribution from the five different crystal domains of the decahedral leg; dashed lines are used to show the manually identified ED pattern from the NMF component #1.

The AuAg NSs studied here display a morphology with a high aspect-ratio (Figures 1 and S2). While the sample contains stars with various leg configurations (see Figure S2), for this study, we have chosen to analyze a symmetrical NS containing 6 legs which can be efficiently produced^3,4^ [see bright-field STEM image (BF) in Figure 1B]. Many studies of Ag nanowires have reported a decahedral structure, with a 5-fold axis along the wire length.^3,17,18^ It is, therefore, not surprising that previous atomic resolution TEM images of NS legs are interpreted as consistent with an elongated decahedral structure containing twins along its axis.^4^ From the crystallographic point of view, a decahedron is formed by the assembly of 5 tetrahedra; nevertheless, a pure face-centered cubic (FCC) structure cannot generate a compact decahedral structure (see Figure S3). Therefore, a structural distortion must occur, what leads to a body-centered orthorhombic (BCO) crystal, as discussed by Yang et al.^19^ and illustrated in Figure S3.

Illuminating information can be derived by evaluating the anticorrelation between the diffraction patterns of each pixel with its neighbors in the 4D-STEM map; these images are brighter at interfaces between phase or grain boundaries.^20^ The anticorrelation image in Figure 1C (and in Figure S18) reveals two points: (i) most legs display bright lines parallel to their axis, revealing the axial twin planes that confirms the decahedral structure of the legs, and (ii) several legs display clear contrast changes along their axis, suggesting a crystal modification, which may be due to growth discontinuities, slight rotation, or mechanical deformation (to be discussed later). Furthermore, the anticorrelation contrast in a cross section of the NS leg is very well described by the expected position of grain boundaries for a distribution of grains around a 5-fold axis, as shown in Figures S11 and S12. Consequentially, the 5-fold symmetry of the legs is confirmed by the reciprocal information (the BCO unit cell measured from the diffraction spot positions) and the real space information (the grain distribution observed by the anticorrelation image is consistent with the 5-fold structure).

There has been much debate on whether this deformation field is homogeneous or inhomogeneous;^18^ here, we will limit our diffraction pattern analysis to a homogeneous BCO structure, as this yields good quality understanding and modeling of our experiments, as described in the Methods section. A schematic drawing of the atomic position within the BCO unit cell and its orientation in a decahedral rod is shown in Figures 1D and S3 (notice that the [010]_BCO_ vector points along the wire axis, Figure 1E).

## Determination of the Star Legs Orientation in Space

The measurement of the 3D leg orientation can be directly obtained by determining the [010]_BCO_ crystal direction (see Figures 1D,E and S3); this is possible by indexing the diffraction patterns as described in the Methods section. A ML clustering tool (*K*-means)^21−23^ has been used to easily group pixels with similar diffraction patterns that spatially corresponded to each leg tip as can be seen in Figure 1F. Note that pixels close to the core or in structurally defective regions are not grouped by the analysis algorithm. To complete the analysis of the whole star, we manually selected regions closer to the core to obtain structural information in the whole leg length, avoiding defective regions (mean ED from tips and bases are displayed in Figures S4 and S5). At this point, the task of estimating the NS shape requires measuring the crystal orientation from merely the average diffraction patterns from 10 different regions (Figures S4 and S5); information on leg lengths can be derived from the images. It is important to consider that each decahedral leg generates a complex ED pattern, as it results from the overlapping of 5 crystal grains. The grains share the same crystal direction ([010]_BCO_) along the leg axis (Figure 1D), but all positioned at different orientations. The initial rough indexing and interpretation of the patterns required manual identification (see Figure S6 for details) of spots arising from one of the five BCO crystals (see examples in Figures 1H and S7–S9). Subsequently, a more precise crystal orientation has been performed with automated crystal orientation mapping (ACOM) numerical procedures to determine each leg axis ([010]_BCO_) orientation with a precision of ∼0.1° (see Figure S10).^24−30^

**Figure 2:**
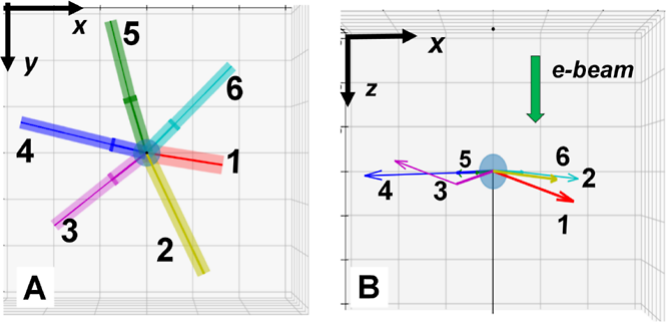
Orientation of the NS 6 legs deduced from ACOM results for bases and tips. (A) Top view, along electron beam direction indicating the legs’ azimuthal distribution. (B) Side view [along the (−*y*) direction] revealing that most of the legs lay in a plane perpendicular to the electron beam direction (the *z* axis points downward in the plot). The background grid indicates 20 nm × 20 nm squares.

Table 1 summarizes the leg orientation in space (vectorial directions), and their spatial distribution is graphically included in Figure 2. Most of the leg bases (#2–6) lay very close to the *xy* sample plane, with the larger deviation observed for LEG#1, which has an elevation angle of about 20.2° (downward in Figure 2). For legs #2 and #4–6, bases and tips show quite similar results with angular changes that do not exceed a few degrees. The tip of LEG#3 points up in Figure 2 showing angular change from the base to the tip of ∼39°, suggesting that this leg suffered a major mechanical deformation when deposited on the grid. In fact, the LEG#3 tip is not free, and it seems in contact with a big particle (see the lower left corner in Figures 1B,C,F and S18). In contrast, for legs #2 and #4–6, the bases and tips show quite similar crystal orientation results with angular changes that do not exceed a few degrees, so we attribute the anticorrelation image contrast changes observed in Figure 1C to instabilities that occurred during growth.

**Table 1:** Spherical Coordinates of the NS Leg Axis (Versors) Derived from Intensity Analysis^a^

leg	base			tip		
	azimuthal [deg]	elevation [deg]	length [nm]	azimuthal [deg]	elevation [deg]	length [nm]
1				9.0	20.2	34
2				64.5	3.6	55
3	142.1	16.5	19	141.5	–22.1	38
4	193.6	2.9	16	193.7	1.9	38
5	253.3	–2.1	24	255.9	0.5	32
6	314.6	1.9	17	314.3	4.9	33

a The azimuthal angle indicates the in-plane (*xy*) rotation of the legs (around *z* axis, starting from *x* axis), while the elevation angle provides the out-plane position (from the *xy* plane, note that in the adopted coordinate system, *z*-axis points down in Figure 2).

To further understand NS formation and the rather planar spatial leg configuration, it is essential to determine the NS core structure because the layout of legs, in first approximation, should be determined by the seed NP structure (or symmetry) used in the two-step NS synthesis procedure.^4,31^

## Determination of the NS Core Structure

**Figure 3:**
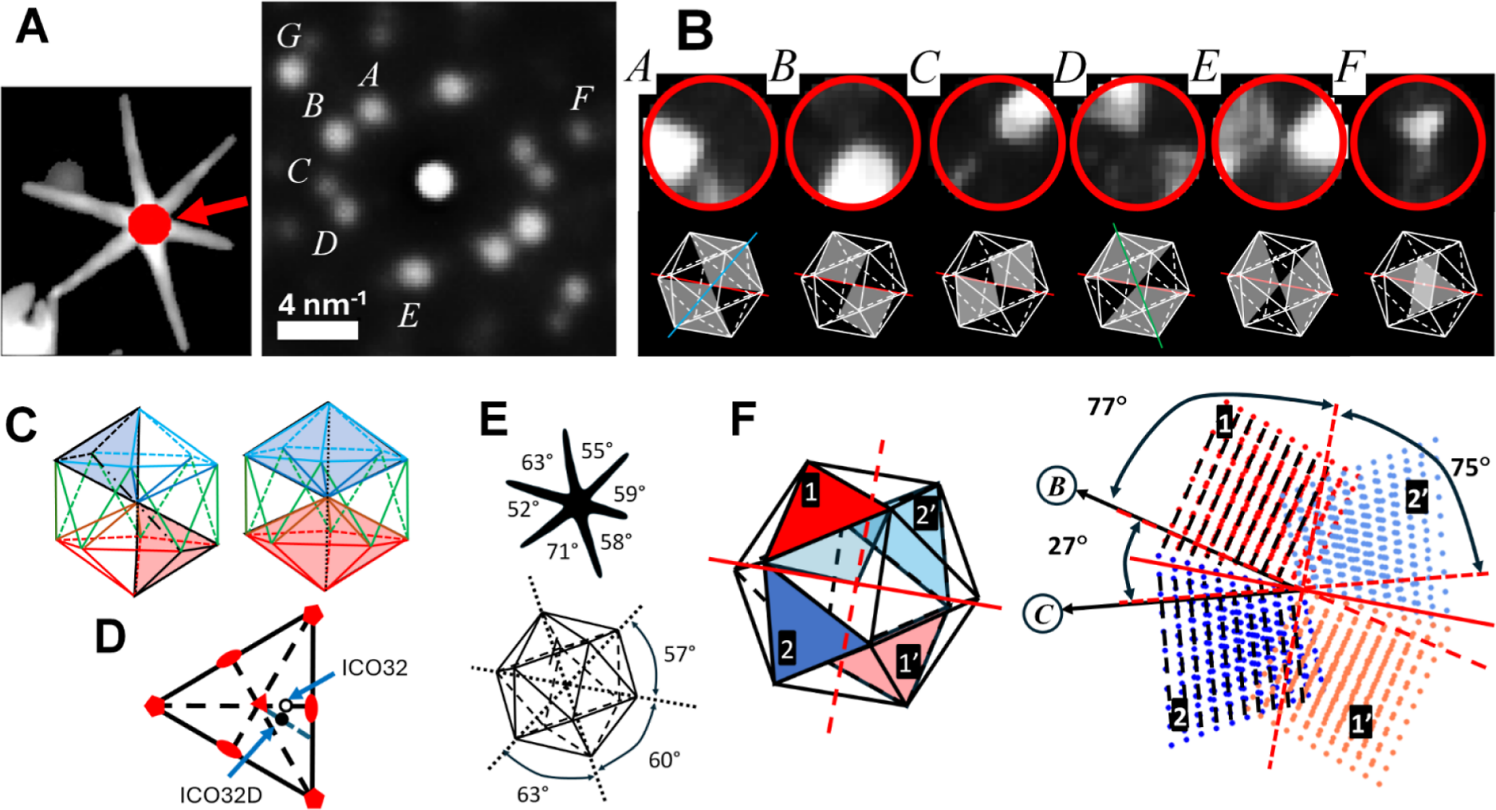
(A) Mean diffraction pattern from the NS core generated by summing all pixels at a distance <10 nm from the estimated core center (shown in the inset). (B) Upper image row: the VDFs derived from diffraction spots marked *A–F* (the circle diameter is 20 nm). Lower image row: the expected VDF contrast generated from the icosahedral geometrical model along ICO32D orientation (the red line in these images indicates the expected twin plane that would agenerate diffraction spot *E* and define the rough 2-fold symmetry of the ED pattern). The simulated VDFs show a very good agreement with the experimental data (in the upper row). Notice that the brighter regions of the VDF from spots *A*, *D*, and *E* are perpendicular to the diffraction peak direction, as expected for regions containing twin planes, providing a solid interpretation for crystal domains generating those peaks. (C) A schematic drawing of icosahedral particles, note that all tetrahedra show a tip at the particle center, and a diametrically opposed tetrahedron rotated by 60°. ICO particles include two stacked decahedra (assembly of 5 tetrahedra) rotated by 36° (2π/10) along the 5-fold axis. (D) The estimated orientation of the NS icosahedral core as observed in the experiments with the incident electron beam impinging vertically onto the page (ICO32D, see the Supporting Information for detailed explanations). (E) The azimuthal distribution of legs derived from NS images in comparison with the position of icosahedral vertices in the estimated ICO core orientation. (F) (left) Schematic drawing of an ICO oriented along ICO32D (see text for explanation). (right) In this orientation, the tetrahedral pairs marked (1 and 1′, 2 and 2′) present {110}_RHO_ atomic planes almost parallel to the electron beam, which would generate strong diffraction spots (marked as *B* and *C*, respectively). This conclusion is corroborated by the excellent match between the predicted and experimental angle and intensity differences between these spots, see Figure S17.

The mean diffraction pattern of the NS core was calculated by adding the contribution from all pixels at the NS central region (10 nm in radius; see Figure 3A). The mean ED pattern shows an elongated hexagonal shape that seems to be roughly close to a 2-fold symmetry with a quasi-mirror plane defined by the line crossing the diffraction spot marked *E* (Figure 3A). VDF images provide the spatial location of crystals generating the different diffraction spots (*A–F*), and we can derive several structural aspects by closely examining these VDFs (Figure 3B): (a) most images reveal crystal regions with a triangular shape at different azimuthal orientation and always showing a sharp tip at the core center and (b) some VDFs show 2 bright triangles that are diametrically opposed and sharing a pointed tip at the core center. These two features can only be explained by a multidomain structure formed by a core with icosahedral symmetry (ICO, see icosahedral schema in Figure 3C), where the noble metal FCC structure is distorted into a rhombohedral (RHO) lattice as discussed by Yang et al.^19^

The experimental ED pattern in Figure 3A shows the characteristics of the 2-fold (ICO2) and 3-fold (ICO3) diffraction patterns of icosahedral particles, such as a rough 2-fold symmetry, but also several diffraction spots (*A*, *D*, and *E*) associated with twin planes visible for ICO3 (Figure S15). Strong evidence of an orientation close to an ICO3 orientation is the generation of spot *F*, which should be observable only for this orientation. Based on the assembled crystallographic data and VDF images, we have been able to conjecture an estimate of the ICO orientation (ICO32D, Figure 3D) very close to the intermediate axis between the 2-fold and 3-fold symmetry axes (ICO32). The deduced ICO32D NS core orientation has been confirmed by the compelling good agreement between simulated and measured diffraction patterns (see detailed analysis in the Methods section and Figure 3).

## Understanding How the Star Core Structure Influences Leg Spatial Configuration

**Figure 4:**
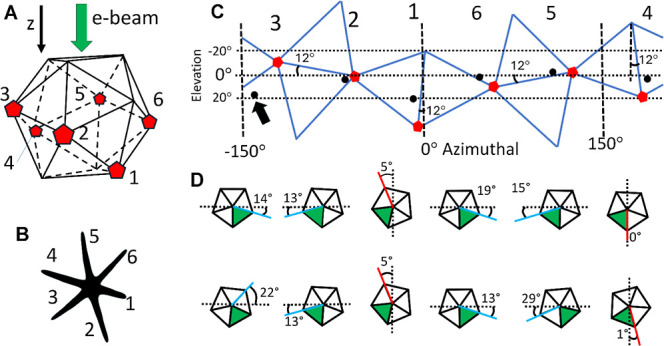
3D correlation between the icosahedral core symmetry and leg orientation. Comparison of the NS measured morphology with the leg azimuthal positions and elevation angle derived from the geometrical NS model. (A) ICO32D core and the expected leg attachment positions at the 5-fold axes located at icosahedral vertices (pentagonal markers), NS legs indexing is shown below in (B). (C) A plot of elevation angle of the vertices as a function of azimuthal angle around the ICO core (pentagonal red markers) compared with measured leg base orientation (round black markers). Note that measured LEG#3 orientation (indicated with a black arrow) is not as close to the predicted position; this is expected as LEG#3 seems to have suffered a major mechanical deformation when deposited on the grid (see text for a more detailed explanation). (D) Orientation of the decahedral leg structure (five crystal domains) around their axis; darkened triangular sectors indicate domains used for leg orientation assessment.

The full understanding of NS atomic arrangement and shape needs the determination of the relation between the NS icosahedral core and leg spatial position and orientation (Figure 4). An ICO particle oriented along an ICO32D axis represents an interesting structure: this configuration presents four corners approximately in the plane perpendicular to the incident beam direction (Figure 4A, expected attachment position for legs #2–3 and 5–6). These ICO corners (actually 5-fold axis) constitute excellent substrates to stimulate the epitaxial formation of decahedral legs close to the *xy* plane (see the geometrical model in Figure 4A,B and angular measurements in Figure 3 and Table 1). This structural model also predicts that two legs (numbered #1 and #4) should grow from apexes that should point 20–30° out of the *xy* plane (upward or downward). LEG#3 data does not follow the previous interpretation (the measured position is indicated with a black arrow in Figure 4C), possibly due to the mechanical deformation mentioned previously. Figure 4 shows a good agreement between the ICO model and the measurement of the legs’ position and orientation, with the small deviations likely related to the imperfect formation of the ICO core.

The ICO vertices represent the ideal 5-fold structured substrate for an epitaxial growth of decahedral wires, so there must be a well-determined register between angular position from the twin planes composing the legs and twins from the core 5-fold axes (Figures 1D and 4C,D). This information is straightforwardly available from the crystal orientation through the measured [0 0 −1]_BCO_ vectors (which are parallel to the decahedral twins). LEG#1 displays the expected twin plane pointing close to the vertical direction from the leg center (upward in Figure 4D). In contrast, LEG#5 (LEG#6) should display a twin laying close to the horizontal plane pointing left (right) from the wire center. Legs #1–3 and #5–6 show a good agreement with this detailed structural model (Figure 4D). Also, we must note that there is a small rotation in the leg structure between the base and tip, suggesting that the legs must include structural defects formed during their growth.

Three capital structural properties of a NS have been determined: (a) the core is an ICO particle; (b) the high-aspect-ratio legs are decahedral rods; and (c) the legs are attached at the vertices (5-fold) of the ICO core. Several previous growth models relate NS shape to a decahedral core, with legs growing from twins perpendicular to 5-fold axis (at 72° between them), and any additional legs (a sixth one) would be located perpendicular to that plane (along the 5-fold axis).^4,31^ In contrast, our results indicate that the 6-leg NS imaged in Figure 1B,C,F grow in an utterly different configuration. Our measurements indicate azimuthal angles between NS legs around 60° (see Table 1 and Figure 3) and an icosahedral core. Previous studies have reported stars with identical branch configuration,^4^ indicating that the derived structural analysis (Figure 1) must be quite general. The occurrence of an ICO core may explain many different NS leg configurations, even when formed by 5, 6, 7, or more high-aspect-ratio legs. Symmetrical 5-branched NS (angle between legs close to 72°, Figure 5g in ref (4)) can be easily accounted for considering 5-fold axes of an ICO core. Some NSs display 5 legs distributed close to the 5-fold symmetry and some additional legs (arrowed NS in Figure 1A or 6c or 6g in ref (4)). These additional legs (sixth, seventh, etc.) are always located close to the central angle between the legs in a 5-fold configuration (see Figures 1 and S23). An ICO particle observed along a 5-fold axis presents 10 tetrahedra sharing an edge with the axis, which can be described as two stacked decahedra which are rotated by 36° (2π/10) in relation to one another (Figures 3C and S23). Thus, the vertices from one of these decahedra support the legs in a 5-fold layout, while the additional legs at bisecting angles must grow in a different plane and from apexes located on the second decahedron (Figure S23).

## Insights into NS Formation during Synthesis

The detailed AuAg NS structural information derived in this work contributes significantly to understanding NS synthesis protocols and growth mechanism. NS synthesis involves two steps: (a) seed formation and (b) a second step to induce leg growth. It has already been observed that it is necessary to increase Ag concentration (from AgNO_3_) during the second step to improve cylindrical shape (reducing tapering) and increase the stability of the high-aspect-ratio legs after growth.^4^ As all legs are decahedral rods, and they contain 5× twins parallel to the leg axis along the whole length. The increase of Ag atoms during leg growth will enhance stability by reducing the energetic contribution of the 5 twin defects (the Ag twin energy is about a factor 2 lower than the Au one^32^). From another point of view, the efficient production of high-aspect-ratio 6-branched NS is favored if the amount of ascorbic acid is raised during leg formation, which is associated with the enhancement of metal atom migration toward low energy facets as {111}.^4^ In fact, the surface at the tip extremes of decahedral wires should be formed by {111} facets; in addition, leg tip surface contains 5 twins which would enhance atom attachment efficiency due to generation of steps and edges during growth. This helps to nucleate new layers, increasing kinetics in a similar way of crystal growth around screw dislocations (Frank growth model^33^).

Our results have revealed that the NS legs located at ICO vertices lay in a highly anisotropic leg distribution (lying very close to a plane, Figure 2B). This contradicts a crucial aspect of icosahedral symmetry: a regular and homogeneous spatial distribution of the 12 corners on a sphere; the ideal star leg configuration on an ICO core would be 12× legs distributed regularly in space. It is expected that the anisotropic growth through seed-mediated methods involving reducing agents, metal salts, and passivating molecules is determined by the relative rate of metal atom deposition and surface diffusion.^34^ However, it seems unlikely that kinetic factors associated with diffusion-limited growth would contribute to induce anisotropy inside the solution. Our hypothesis is that, as the NS core is formed during the first synthesis step (i.e., seed generation), the nucleation and further growth of the legs during the second synthesis step may require the unzipping of the surfactant layer on the NP.^4^ Upon nucleation of the first leg on an icosahedra vertex, the surfactant layer might be disturbed, rendering the nucleation of a second leg on a neighboring apex easier. The nucleation at two neighboring apexes may be able to generate a linear crack in the surfactant layer, facilitating its further unzipping along the expanding unzipping line. Simultaneously, this may increase the pressure on the direction perpendicular to the plane defined by the unzipping line on the core; as a result, the nucleation of the out-of-plane legs could be inhibited. In this scenario, the anisotropic leg distribution could be due to an interplay between the symmetry of the icosahedral core and the crack line on the surfactant capping layer. As this is only a working hypothesis of the complex and currently unknown mechanistic events taking place during the growth of these NSs, more detailed experimental work is necessary, and it is currently the focus of our synthetic efforts.

## Conclusions

The precise crystallographic analysis reported here provided a complete characterization of the NS 3D crystalline structure. We must emphasize that all structural measurements have been confirmed by the analysis of a second 4D-STEM scan with the sample holder rotated by 10°; also, this second data set provided evidence of no detectable structural damage, confirming the low dose utilized in the experiment.

We speculate that the measured NS shape and legs’ spatial distribution is associated with the surfactant layer unzipping over the icosahedral core during growth. It is essential to continue to refine the chemical and structural characterization of products at different stages of the complex two-step synthesis protocol to improve the understanding. This would be fundamental for NS synthesis scale-up and technological application.

Once we have a detailed measurement of the 3D shape, it is crucial to apply this knowledge to develop more precise modeling of their optical response and a deeper understanding of their application as plasmonic antennas.^35−38^ The optical response results show a very good agreement with the measured spectrum for an ensemble of NS, providing more detailed understanding of their optical properties (see Figures S24−S26).

Determining the 3D morphology of a NS represents a traditional and well-stablished application of nanometer-resolution ET; in fact, HAADF ET could have been applied as an independent verification approach. However, the derivation of atomic arrangement information from higher resolution ET experiments remains quite challenging for this kind of extended NPs. Notwithstandingly, tomography image reconstruction is more suitable for nanomaterials which are not beam sensitive, where sample thickness and height (inducing defocus) do not change significantly during the tilt series: NPs, nanorods, nanocubes, etc., within the suitable dimension size (<20 nm). This study has provided solid evidence that we have been able to derive a very detailed atomic arrangement (legs, core, and the relative spatial orientation) from an individual very complex multibranched metal plasmonic NP from a single 4D-STEM scan, which was acquired using a very low electron total dose (<200 e^–^/Å^2^ per frame). The utilized analysis is especially well suited for the characterization of objects with extended and asymmetric shape. Our study has been based on ED techniques, which demand a more complex data analysis than the intuitive visualization of a tomography 3D image. The procedures applied in our work and results obtained provide opportunities to determine the atomic structure of individual multidomain or/and multiphase NPs, even when constituted of beam-sensitive materials.

## Methods

### Specimen Preparation

The Au seed NPs were initially synthesized by the addition of a freshly prepared ice-cold solution of NaBH_4_ (0.6 mL, 0.01 M) into an aqueous solution of HAuCl_4_ (10 mL, 0.25 mM) and Triton X 100 (0.15 M). The solution immediately turned from pale yellow to orange after the addition of NaBH_4_. The mixture was stirred for 2 min and aged for 10 min at 4 °C before use. The star growth solution was prepared by adding 0.4 mL of 25 mM HAuCl_4_ solution to a 20 mL Triton-X solution (0.15 M). This step was followed by the addition of ascorbic acid (1.2 mM), AgNO_3_ (100 μM), and the Au seeds (ranging from 0.06 nM) to the growth solution. The solution was stirred for 10 min and then centrifuged at 3500*g* for 10 min and dispersed to a final concentration of approximately 2 nM with Ultrapure Milli-Q water (18.2 MΩ cm). Samples of the produced 6-branched NSs with high-aspect-ratio legs were then drop-cast on a holey carbon Cu grid (Figures 1 and S2).

### Electron Microscopy Experiments

A Thermo Fischer Spectra 300 electron microscope operated at 300 kV equipped with a probe spherical aberration corrector and a Quantum Detector Merlin 256 × 256 pixels direct detection camera was used for recording STEM and 4D-STEM data. Precession ED (PED) at 1 kHz with a precession angle of 1° was generated using a Nanomegas hardware and TopSpin software. Data acquisition used a 2 nm-wide electron probe of 1.0 mrad half-convergence angle and a dose of <200 e^–^/Å^2^ per frame; the pixel step was 1 nm, and the dwell time was 1 ms. Low-resolution study of the sample was based on a 200 kV using a Topcon 002B TEM. More detailed description of the PED experiment can be found in the Supporting Information.

### Calculation of Virtual Images (VDFs) from 4D-STEM Maps

The interpretation of virtual images was fundamental for this work; thus, it was important to understand the contained spatial information with the maximum possible precision. Different kind of images has been calculated: (a) VADF, where the intensity in annular areas of the diffraction patterns (including many diffraction peaks) is added to generated images, or (b) VDF by selection of individual diffraction peaks to generate specific sample regions associated with a particular diffraction spot (see Figure 1).^13,15^

We have utilized anticorrelation images to visualize grain distribution in the NS; they were constructed with the common definition of anticorrelation utilized in 4D-STEM work, which compares a diffraction pattern *p*_*x*,*y*_ (*x*,*y*: spatial coordinate in the ED mapping) with its nearest neighbors (*p*_*x*+1,*y*_ and *p*_*x*,*y*+1_) to form contrast *C*(*x*,*y*)^20^
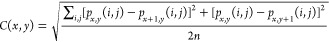
1where *i*,*j* is the coordinate of a pixel in the measured ED, with *n* total pixels in each one.^20^ The value of *C*(*x*,*y*) is low when neighboring pixels show similar diffractions patterns and high when patterns change significantly from pixel to pixel; this is a very efficient tool to identify crystal grain boundaries and twins (see Figure 1).

### Details of 4D-STEM Diffraction Data Processing and Simulation

The 6-branch star is quite an open nanostructure, in which the area that it occupies (a circle with a diameter of ∼120 nm, the distance from a leg tip to the opposite one) is much larger than the effective projected area that the NS fills. Then, most pixels in the original 200 × 200 map just contain information from the carbon substrate (amorphous), not the NS (crystal). To reduce data volume, we have binarized a VADF image and selected all bright pixels to build a reduced data set containing just the pixels (∼3200) inside the 2D projection image of the NS (a 92% reduction in data size). ML tools were applied using the Hyperspy open package.^23^ Intensity profiles were derived using the ImageJ free software.^39^ Orientation analysis was based on the Pyxem open software,^24^ and PED intensity analysis has been previously developed and applied using a homemade Python software.^29,30^

### Background Subtraction

The data block has been processed to reduce the effect of nonspecific (substrate or inelastic) intensity. A simple background subtraction was applied: a threshold was utilized in the VADF image to separate crystalline (inside NS) and amorphous (carbon substrate) regions. A background mean diffraction pattern was utilized as a template, which was rescaled for each pixel as a PED background and finally subtracted.

### Clustering and ML

Clustering has been applied to our 4D-STEM diffraction maps by utilizing algorithms available in the Hyperspy suit,^23^ whose efficiency has already been well established for treatment of diffraction data.^21,22^ A gamma function has been applied to enhance the influence of low intensity peaks for ML algorithm processing, and a threshold value was utilized to exclude peaks with excessively low intensity. The *K*-means method identified 8 clusters, which resulted in 6 clusters spatially localized in the NS leg tips, and the other ones displayed mixed contribution of the NS core and the leg bases. Only LEG#1, highlighted in red in Figure 1F, could be fully clustered from the core to the tip by ML (the mean ED pattern from this region is shown in Figure 1G); this leg seems to be different from the other 5 legs, being shorter (∼30 vs 50 nm) and slightly wider (12–15 vs 7–10 nm). We have also selected regions close to the NS core (indicated as base regions, see Figure S5) to ensure the characterization of the whole leg. The mean diffraction patterns of tip and base regions have been calculated (see Figure 1 main text and Figures S4 and S5) and analyzed in the same way. This procedure has drastically reduced the number of diffraction patterns that can be interpreted to just 10 patterns in total.

For each leg, the final step of interpretation of crystal orientation involved the manual recognition of the geometrical pattern of spots arising from one of the five BCO composing the decahedral leg; this allowed us to perform a rough crystal indexation with the common procedure (see Figure S6 for an example) to ensure the reliability of the crystal identification. When manual identification was rather difficult, we have also applied NMF (from Hyperspy^23^) to a data block generated by pixels in each individual region grouped by clustering (no intensity modification procedure, as scaling and gamma function, has been utilized here). The NMF was not able to isolate or recognize each of the 5 crystals forming the decahedral leg; nevertheless, it was useful in a few cases (LEG#1) to render easier the subsequent manual interpretation of diffraction spots (see Figures 1H, S6, and S7).

### Crystal Orientation Determination

The use of conventional template matching is the most straightforward method to measure crystal orientation; however, the complexity of our sample implied the need of a more robust approach.^24−26^ The polycrystalline nature of the legs (diameter <15 nm) implied that the observed diffraction pattern results from the contribution of several superimposed crystals (5, each with its own zone axis); this leads to an ineffective automated determination of crystal orientation using the standard procedures. Therefore, an additional manual identification protocol has been required, which can be performed by the traditional indexation of ED patterns.

We must identify diffraction spots from one of the 5 crystal domains forming the decahedral leg, so they can be selected by using a series of masks (shown in Figure S9). After masking, a gamma function is utilized to enhance the influence of diffraction peaks with low intensities; then, spots above a threshold value are selected to generate a binarized diffraction pattern. We have utilized the Pyxem software^24^ to perform template-matching ACOM and retrieve the [010]_BCO_ and [001]_BCO_ directions. The template library has been simulated for a Au BCO crystal with the primitive cell dimensions measured from the diffraction pattern and calibrated from a Si calibration sample in the same experimental conditions (*a* = 0.265 nm, *b* = 0.271 nm, *c* = 0.358 nm, space group = *Immm*, #71). It is important to notice that the indexation can be performed with high precision due to the capacity to differentiate between the three main axes of the cell (*a*,*b*,*c*). The *c*-axis ([001]_BCO_) is significantly distinct from the *a*-axis ([100]_BCO_) and (*b*-axis [010]_BCO_), and we can differentiate the *b*-axis by observing the direction of the leg in the virtual images formed by the diffraction patterns. In general, ACOM is very reliable in less symmetrical crystals, for example, the orthorhombic or hexagonal crystals, which simplifies the identification of a specific direction by reducing the multiplicity of each direction.

Pattern-matching ACOM results indicate that most leg axes ([010]_BCO_) are confined to the *xy* plane, and the only major difference in orientation is the azimuthal angle of each one. This loss of some 3D information is probably the consequence of the binarization of intensities, as usually applied in pattern matching algorithms.

The diffraction spot intensity provides precise 3D information, as observable in the intensity profiles. For example, looking at extracted profiles from LEG#2 along the leg axis (Figure S4, left to right follows the core to tip direction), we can notice that diffracted beams on the left (core) side of the transmitted beam display higher intensity than beams on the right (tip) side. Then, the Ewald sphere construction and the Laue circle in diffraction physics^40^ easily explains this intensity difference, what unambiguously indicates a leg oriented with its tip moving (down) along the electron beam traveling direction. Notice that the sample coordinate system indicated in Figure 2 implies in a e-beam traveling along the *z* direction (downward), a common coordinate reference system utilized in ACOM softwares.^24−26^ We would like to mention that LEG#1 displays an elevation angle of 20° so that it has not been possible to exploit Laue circle construction to determine if the leg is pointing downward or upward (phenomenon described as 180° duality of SAED). Then, a second diffraction map (Figure S18) taken at a different incident angle to the NS plane has been necessary to determine unambiguously that this leg points downward.

First, we obtain crystal orientation from pattern-matching as a starting point, where the angular resolution is at best limited to ∼1–2° (see Figure S9); this is a common value for methods based on cross-correlation between experimental patterns and a library of kinetically simulated ED patterns.^24−26,29,30^ Subsequently, a second higher precision ACOM was applied, in which the quantitative analysis of diffraction beam intensity is exploited (for this step, we used raw intensities after background subtraction, without any further intensity modification); the crystal orientation is measured through a residual metric (Rietveld-like comparison of experimental and simulated beam intensity, as illustrated in Figure S6) that allows angular resolution to be improved by at least 1 order of magnitude. We estimate that crystal orientation angles have been determined with a precision of ∼0.1° (see Figure S10). The quality of the optimization is assessed by the residue value,^29,30^ our results indicate the value in an acceptable range (20–40%),^41^ considering the complexity of the NS structure and the unavoidable overlap between diffraction peaks from different crystal domains in the legs.

Overall, measurement of crystal orientation of the NS legs is a challenging case for both the peak position based and the intensity-based methodologies. The small crystal thickness (at maximum ∼10 nm) implies large excitations errors, and peaks can be excited even with a large disorientation (<5°) from ideal diffraction condition, this is evidenced by the high symmetry of the measured PED patterns (see Figures S8 and S9). This probably implies that template matching cannot identify changes in orientation in this rather large angular range. This is corroborated by our results, where template matching provided many NS leg orientation restricted to the *xy* plane (0° elevation angle), but using diffracted intensity analysis intensities, we have revealed *xy* plane disorientation of the legs up to 5° (see Table 1). Furthermore, quantitative analysis of intensities must be performed with care as many of the observed PED peaks are resulting from the superposition of reflections from different crystals, affecting proportions between diffracted peaks in unpredictable ways. Consequentially, residues are higher than expected for a single crystalline case, and the angular resolution (∼0.1°) is higher than previously reported efforts (<0.05°).^29,30^

### NS Core Structure

Noble metal NPs can form different types of so-called multiple-twinned particles (MTPs) including a 5-fold axis; the decahedral structure is one of them, and its orthorhombic atomic arrangement has been extensively discussed to analyze leg structure (Figure 1D). The second type of MTPs is the icosahedron, frequently observed in Au or Ag NP samples.^19,42−44^ An ICO is formed by the assembly of 20 tetrahedra sharing the tip at the center (Figure 3C). To fill the space, the cubic FCC unit cell is compressed along the [111] direction to generate a rhombohedral unit cell (RHO, space group = *R*32, #155, *a* = 0.289 nm).^19^ From the point symmetry, ICO particles present 6× 5-fold axes (located at corner), 10× 3-fold axes (center of triangular faces), and 15× 2-fold axes (center of edges).^19^ There are many reports of atomic resolution images of ICO NPs along 2- and 3-fold axes (hereafter noted as ICO2 and ICO3 respectively), and image contrast may be rather complex, but they can be easily understood considering some tetrahedra oriented correctly along the crystal zone axis (Figure S15).^43,44^ The diffraction pattern originated by a system of 20 crystalline domains has revealed to be too complex to be solved by our manual analysis or by trying to unmix information by ML methodologies. Thus, we have not been able to calculate precisely the ICO orientation directly from the diffraction pattern as previously realized for the decahedral legs (see Table 1). However, when an ICO is oriented along an axis at the intermediate direction between a 2-fold and a 3-fold axis (an angular distance of 10.5° from both axes, Figure S16), the diffraction spots should display an elongated hexagonal configuration as observed in our experiments, in agreement with the analysis by Reyes-Gasga et al.^43^ (see more details in the Supporting Information). Furthermore, an icosahedron oriented as ICO32D would display some tetrahedral units (marked 1, 1′ and 2, 2′ in Figure 3F), which will present atomic planes almost parallel to the electron beam and that will generate strong diffraction spots (*B* and *C*). From that, we can determine an ICO slight outside the intermediary orientation by looking at the projected atomic positions of these pairs of tetrahedra (Figure 3F). Notice that in this case, 2,2′ crystals are slightly more misoriented (3° off) from the electron beam direction than 1,1′ crystals. Consequentially, diffraction beams associated with 2,2′ tetrahedra will show lower intensity than the ones from the 1,1’; our model predicts that spot *C* in Figure 3F should be weaker than spot *B*; this is in full agreement with experimental data (simulated diffraction patterns is shown in Figure S17).^45^ If the incident direction is along the line connecting the 2-fold and 3-fold axis, the intensity of spots *B* and *C* should be alike (both crystal pairs become nearly identically oriented along the e-beam direction, Figures S13 and S21).

### Verification of Robustness and Accuracy of the Structural Results

To analyze the reproducibility of the applied procedure, we have acquired an additional data set by tilting the TEM sample holder by 10° and, subsequently, performing identical data processing and analysis. Unfortunately, the second 4D-STEM data set shows significant effects of amorphous carbon deposition after acquisition of data at 0°; thus, the quality of recorded diffraction data was lower (see Figure S18). Usually, this level of contamination would represent a serious difficulty for atomic resolution imaging; however, ED is a much more robust methodology to reveal atomic arrangement information under these sample conditions. The anticorrelation image from the data set taken at 10° (Figure S18) reveals easily that all twins associated with the decahedral structure of the legs have been conserved, confirming the low-dose profile of the present study (no radiation damage effects have been detected). More importantly, all of the crystallographic conclusions obtained from the second experiment (see details in the Supporting Information, Figures S18–S22) fully agreed within experimental error with the original study, confirming the leg spatial distribution and the icosahedral structure of the NS core and its estimated orientation.

### Simulation of the Optical Response

The electromagnetic extinction calculated through the discrete dipole approximation^46^ qualitatively agrees with the optical characterization of the sample (see Figures S24 and S25). Below 600 nm, the optical responses in both experimental and simulated extinction spectra are very similar and are dominated by the plasmonic response of the gold spherical core (∼520 nm) and interband transitions (background). Two broad resonances are observed in the experimental data at 670 and 830 nm. In the simulated spectrum, we were also able to observe two resonances, namely, mode#1 (∼630 nm) and mode#2 (∼750 nm). The observed resonances are blue-shifted and considerably narrower than the experimental observations. These discrepancies can be attributed to different factors: (i) the real sample is composed of a distribution of NP shapes, whereas only one structure is considered in the simulation,^47−49^ (ii) the simulated structure must yet be improved to reproduce a nanostructure with such a diversity of intricate features, (iii) possible local refractive index effects due to molecular adsorbates from the synthesis were not included, (iv) a possible difference in composition for the legs, containing non-negligible Ag, was also not considered. Nevertheless, the observation of two modes in the simulated spectrum is an important realization and is in good qualitative agreement with the more complex experimental spectrum. The polarization vectors for each dipole are presented in Figure S26 and suggest that mode#1 is composed of dipole oscillations localized mainly on 2 legs (LEG#2 and LEG#6) for the incident polarization, whereas in mode#2, the contribution of four legs can be observed (LEG#2, LEG#3, LEG#5, and LEG#6). The results suggest a high degree of plasmon resonance and near-field tunability by controlling the number and aspect ratio of legs in AuAg NSs.

## Data Availability

Supporting Information is available in the online version of the paper. The data sets utilized in this work is available at https://redu.unicamp.br/dataset.xhtml?persistentId=doi:10.25824/redu/YYUUDW. This data is registered with the DOI: doi:10.25824/redu/YYUUDW.

## Supporting Information Available

TEM micrographs of the sample; operational principles of 4D-STEM diffraction mapping and precession electron diffraction; atomic arrangement of decahedral particles; clustering (*K*-means) grouped pixels, the resulting mean diffraction patterns and diffracted intensity profiles; manual identification of zone axis and crystal orientation; mean diffraction pattern of LEG#1 and the result of NMF analysis; manual identification of the zone axes for LEG#1 and crystal orientation with template-matching; quality, sensitivity and precision of the intensity analysis through residue minimization; evidence of 5-fold symmetry of the NS legs; mean diffraction pattern for the NS core; structural aspects associated to ICO particles structure; deduction orientation of the NS icosahedral core; simulation of the NS core diffraction pattern; VADF and correlation-image of the NS after sample rotation; determination of the rotation axis and NS 3D reconstruction and core orientation after sample rotation; measured PED pattern; ;icosahedral core for NS showing 5-fold symmetry in leg orientation; UV-Vis-NIR spectra, DDA-simulated extinction spectra and polarization vectors of the derived structure (PDF)
