# Supplementary material for: Atomic Structure and 3D Shape of a Multibranched Plasmonic Nanostar from a Single Spatially Resolved Electron Diffraction Map
Source: ACS Nano. 2024 Sep 21;18(39):26655–65. doi: 10.1021/acsnano.4c05201 (PMC11447907; doi:10.1021/acsnano.4c05201)
Supplement: Supplementary file 1 — nn4c05201_si_001.pdf [file nn4c05201_si_001.pdf]

Supplementary Information

Atomic structure and 3D shape of a multi-branched plasmonic nanostar from a single spatially resolved electron diffraction map

Leonardo M. Corrêa[a], Simon M. Fairclough[b] , Kaleigh M. R. Scher[c] , Supriya Atta[d] , Diego Pereira dos Santos[e], Caterina Ducati[b], Laura Fabris[f], Daniel Ugarte[a*]

[a] Instituto de Física Gleb Wataghin, Universidade Estadual de Campinas, Campinas, 13083- 859, Brazil.

[b] Department of Materials Science and Metallurgy, University of Cambridge, Cambridge, CB3 0FS, UK.

[c] Department of Materials Science and Engineering, Rutgers University, Piscataway, NJ 08854,USA.

[d] Department of Biomedical Engineering, Duke University, Durham, NC 27708, USA.

[e] Instituto de Química, Universidade Estadual de Campinas, Campinas, 13083- 859, Brazil.

[f] Department of Applied Science and Technology, Politecnico di Torino, Turin, 10129, Italy.

* Email: dmugarte@ifi.unicamp.br

Precession electron diffraction (PED)

The interpretation of electron diffraction (ED) is rather complicated due to the strong electron-matter interaction (a phenomenon described as dynamical diffraction) that leads to nonlinear effects on the diffracted beam intensities such that the data can only be correctly modelled through slow and complex numerical simulations (*1*-*3*).

Vicent & Midgley (*4*) have proposed a method of precession electron diffraction in which the ED pattern is acquired while the electron beam precesses around the microscope optical axis (forming a hollow cone, Fig. S1). This beam manipulation reduces dynamical diffraction effects, such that beam diffraction patterns can be quantitively understood using a simpler x-ray crystallography modelling, which is the so-called kinematical theory (*4*-*8*). Scanning precession electron diffraction (SPED) is also widely used in materials science for texture analysis by means of the so-called automated crystal orientation mapping (ACOM) (5,*9*,*10*). We have obtained a higher precision measurement of crystal orientation by a quantitative analysis of diffracted beam intensities (*11*,*12*), which will be discussed in more detail in the crystal orientation determination section.

The use of PED implies that events of double scattering will be significantly reduced such that, in the case of crystal superposition, we can analyze the final diffraction pattern as the incoherent addition of each crystal scattering (each crystal can be treated as isolated). This has been exploited for crystal tomography and the reconstruction of precipitates in superalloys based on PED diffraction mapping by Eggeman et al. (*13*).

Determining the NS core atomic structure

The core mean diffraction pattern has been obtained by averaging all patterns from the nanostar (NS) centre (circular region of 10 nm in radius, Fig. 3 main text and Fig. S13). This pattern shows an elongated hexagonal distribution of spots, roughly close to a 2-fold symmetry.

The spot distribution reminds a face centred cubic crystal (FCC) crystal containing 2 non-parallel twins (Fig. S14) as described in the transmission electron microscopy (TEM) textbook by De Graef (*19*). Nevertheless, this explanation can be easily ruled out by taking virtual dark field (VDF) images of diffractions spots in our experiment (Fig. 3 main text), which are quite different from the expected VDFs image contrast for FCC twinned crystal (Fig. S14). Looking carefully at VDFs from the NS core (Fig. 3 main text), we can recognize that most VDFs reveal crystal regions with a triangular shape at different azimuthal orientation and always showing a sharp tip at the core centre. These characteristics can only be explained by an icosahedral structure (ICO, see geometrical schema at Fig. 3), with rhombohedral (*RHO*) atomic arrangement.

The roughly 2-fold nature of the core mean ED pattern (Fig. 3 and Fig. S13) suggests an icosahedral particle observed along 2-fold axis (ICO2 observation). However the pattern also shows very clear attributes of an ICO3 (Fig. S15): a) an angle of 60 degrees between spots marked D and E ($(110)_{RHO}$ planes, Fig. S13); and b) the occurrence of the spot marked F ($(\bar{1}10)_{RHO}$ or $(121)_{RHO}$ planes, or {220} planes in the undeformed FCC lattice, Fig. S13) aligned with the same direction as spot D. The interplane distance associated with spot F is much smaller (~1.4 Å) than the ones originating A-D spots (~2.2 Å, see Fig. S13), so these planes require a much more precise crystal orientation to be measured (expected diffraction patterns from ICO2 and ICO3 are displayed in Fig. S15). The simulated VDF for spot F shows a bright contrast at the particle centre in full agreement with the experimental VDF image expected for an icosahedral particle observed along 3-fold axis (ICO3 observation) (Fig. 3 main text).

Unfortunately, we have not been able to calculate precisely the ICO orientation directly from the diffraction pattern as previously realized for the decahedral legs (see Table 1). The experimental ED shows characteristics of the 2-fold and 3-fold diffraction pattern. The intermediate direction between a 2-fold and 3-fold axis (ICO32, noted 32 in Fig. S16) at angular distance of 10.5 degrees from both axes. To preserve a good orientation of atomic planes originating the diffraction spot marked F, the ICO must be rotated along a direction close to normal to these planes (see lower part of the Fig. S16) in such a way that the 2-fold nature of the diffraction pattern is preserved. We have conjectured a possible observation direction to fulfil all experimental constrains (arrowed in Fig. S16), which will be called ICO32D; letter D is included to describe a deviation for the intermediate position (ICO32) between ICO2 and ICO3 orientations. It is important to note

that an icosahedron along the ICO32D orientation yields a complete consistent explanation for all experimental data (VDF images in Fig. 3) and predicted diffraction spots, see Fig. S13 and S17).

Taking ICO32D orientation as the reference, we can geometrically model the orientation of different tetrahedra forming the ICO core, estimate their diffraction pattern and their spatial location through VDF images. Some domains (marked 1,1' and 2,2' in Fig. 3F) will present atomic planes almost parallel to the electron beam and should generate strong diffraction spots. Fig. 3F shows the projected atomic positions of these tetrahedra; note that tetrahedra 1 and 1' display a family of $(110)_{RHO}$ planes very well oriented along the electron beam direction generating a strong diffraction spot (disk marked *B* in experiment). In contrast, domains 2 and 2' display a similar family of planes that are, however slightly misoriented (misorientation of about 3 degrees with the electron beam), which would generate much weaker spots (disk *C*). The predicted spot position, intensity and their relative angle is in excellent agreement with measurements (see Fig. S13). Concerning the intense spot *G* (not shown), its VDF image does not follow a contrast pattern that can be associated with an ICO core; we attribute its occurrence to a region of metal overgrown on the ICO seed during the NS leg growth and it will be neglected in our analysis.

We must emphasize that we have been able to predict the spot positions, angular distance, and intensity differences from the ICO23D model (see Fig. 3, Fig. S13 and S17). Briefly, a precise understanding of the NS core atomic arrangement has been derived by the combination of electron diffraction and virtual images. All major diffraction spots (*A*-*F*) from the NS core have been explained through well founded arguments.

<u>Verification of the robustness and accuracy of the structural results</u>

The crystallographic analysis of ED patterns may seem rather difficult, and thus it may raise questions and doubts on the robustness of the procedures. To provide an answer to the potential questions on precision, accuracy, and reproducibility, we have used the tilting capacity of TEM sample holders to acquire a second set of data after rotation of 10 degrees. Subsequently, we have applied the same data analysis procedures. Unfortunately, the quality of recorded diffraction data resulted much lower due to a significant deposition of carbon contamination (see Fig. S18), which reduced the precision of derived structural parameters. It is important to emphasize that the NS images taken at 0 and 10 degrees do not show major difference, but ED data from legs or the NS core show major changes (Fig. S19 & S21). Fig. S18c displays the anticorrelation image after 10 degrees tilt, where twins from the leg decahedral structure are clearly visible (bright lines along legs) implying that the decahedral atomic arrangement has been conserved, and thus confirming the low dose profile of this study.

To test the quality of our crystal orientation assessment, we have compared first and second assessment of leg spatial position considering the applied 10 degrees rotation. We have analysed LEG#4 and LEG#6 which should show the maximum orientational changes as these two legs are located at high angle (closer to perpendicular) from the sample holder rotation axes (approximatively along vertical direction). The $[010]_{BCO}$ direction has been determined for these legs: a) LEG 4 = [-0.965, -0.221,-0.143]; b) LEG 6 = [0.644, -0.7352, 0.222]. The rotation axis (dashed line in Fig. S19) has been determined by optimizing the azimuthal angle to the axis, such that experimental measurements result from an applied rotation angle of 10 degrees. The angle between legs ($\alpha_{4\text{-}6}$) has determined by using the scalar product between the vector indicating the axis of the decahedral structures by using the crystallographic analysis of the experimental PED patterns. The results show full agreement within experimental errors: a) 0 degrees experiment $\alpha_{4\text{-}6}$= (120.3 ± 0.1) deg.; b) 10 degrees experiment $\alpha_{4\text{-}6}$= (119,4 ± 0.4) deg. Furthermore, data quality (ex. presence of saturated diffraction spots) has hindered the analysis of diffraction intensities, so we have perform a simpler orientation analysis for the other legs (reduced angular precision in the few degrees range). The results are shown in Figure S20 and Table S1 and confirm unambiguously the planar leg configuration. However, the angular resolution for legs 1, 2, 3 and 5 is at best limited to the resolution provided by PYXEM (~ 1°), which is optimistic considering that sample characteristics (mainly the sample thickness, ~ 5 nm of crystallite size) restrict the methods (only peak position is taken into account). This is evidenced in LEG#1, where we have only been able to identify only a major axis ($[-2\ -1\ 1]_{BCO}$), a deviation of 5 degree from the expected axis ($[-8\ -5\ 4]_{BCO}$) calculated from the measured axis of the original data. Consequentially, we decide to utilize a 5 degrees value as a conservative

estimation of the angular resolution. Although the reduced precision, it is still to corroborate the planar leg configuration obtained with the original data (before carbon contamination occurrence).

Table S1: Spherical coordinates of the NS leg axis (versors) derived from the intensity (Leg#4 & Leg#6) and with template-matching (1, 2, 3 & 5) have distinct angular resolution. The table follows the same conventions than Table 1 in the main text.

Leg	Azimuthal [deg]	Elevation [deg]
1	9.6	28 ± 5
2	66.1	9 ± 5
3	140.6	18 ± 5
4	192.9	-8.2 ± 0.3
5	253.2	-7 ± 5
6	313.2	12.8 ± 0.2

Concerning the NS core, we evaluated the mean ED pattern from the central region (Fig. S21) from the 10 degrees rotated NS. Surprisingly, the general appearance of the pattern is very similar to the unrotated sample one, showing again an elongated hexagonal distribution of spots roughly close to a 2-fold symmetry, but the mirror plane seems rotated azimuthally. This is due to a change of the ICO32D icosahedral to another 2-fold axis surrounding the central point of an icosahedral triangular facet (see Fig. S21b). This result is not surprising because the icosahedral point group shows high density of high symmetry axes. The mirror dividing the hexagonal configuration of the diffraction pattern follows the plane determined by spot marked *T*. Spot marked *U* is associated to $(10\bar{1})_{RHO}$ planes that should observed when an icosahedron is oriented along ICO3 axis. As spots *T* and *U* are aligned along the mirror plane, we must conclude that the ICO orientation is located approximatively along the line connecting 2-fold and 3-fold axes. This is confirmed by the fact that diffraction spots *S* and *R* show almost identical intensity (equivalent spots in the original ICO32D orientation were marked *B* and *C*, but they showed quite different intensity). The geometrical reconstruction of an icosahedron confirms this analysis (compare Fig. 3 and Fig. S21 & S22). Tetrahedra originating both diffraction spots should show atomic planes along the electron incident direction and will generate intense electron diffraction spots of similar intensities. Additionally, the measured and expected contrast of VDF generated from *S* and *R* spots show a remarkable agreement.

Summarizing, all the crystallographic conclusions from the second experiment fully agreed with the original study, confirming leg spatial distribution and the icosahedral structure of the NS core and its estimated orientation (ICO32D).

<u>Analysis of 6-branched or 7-branched NSs showing 5-fold symmetry.</u>

The results showed unambiguously that the core of the 6-branched NS is an icosahedral nanoparticle; however, previous reports indicate that the core is more akin to decahedral. This is mostly based in the fact that the legs of many NS follow a 5-fold layout: 5 legs nucleate with a 72 degree between neighbouring legs which are correlated to the decahedra 5 twin positions. An alternative explanation can be derived from the present work that demonstrated unambiguously an icosahedral particle as the NS core. An ICO particle observed along a 5-fold axis presents 10 tetrahedra with an edge parallel to the 5-fold axis. These 10 tetrahedra can be grouped and described as two stacked decahedra; the two decahedra are rotated by 36 degrees ($2\pi/10$) in relation to one the other (Fig. 3C and Fig. S23). Thus, assuming that the legs in a 5-fold layout are, all the five, located on the vertices of one of these decahedra, the additional leg at bisecting angles (36 degrees) must grow in a different plane and from apexes located on the second decahedron. Fig. S23 shows a TEM image of a particle (arrowed in Fig. 1A) displaying 5 legs azimuthally distributed close to 5-fold symmetry and a 6th leg (located close to the central angle between two legs defining the 5-fold symmetry). This leg configuration represents also a layout expected for an icosahedral NS core (see Fig. S23).

Absorption measurement and simulation

Absorption spectra were collected on a Thermo Scientific Evolution 300 UV-Visible spectrophotometer using a quartz cuvette with a 1 cm path. Simulated extinction spectrum for the measured nanostar 3D morphology has been calculated using DDA approach (*15*).

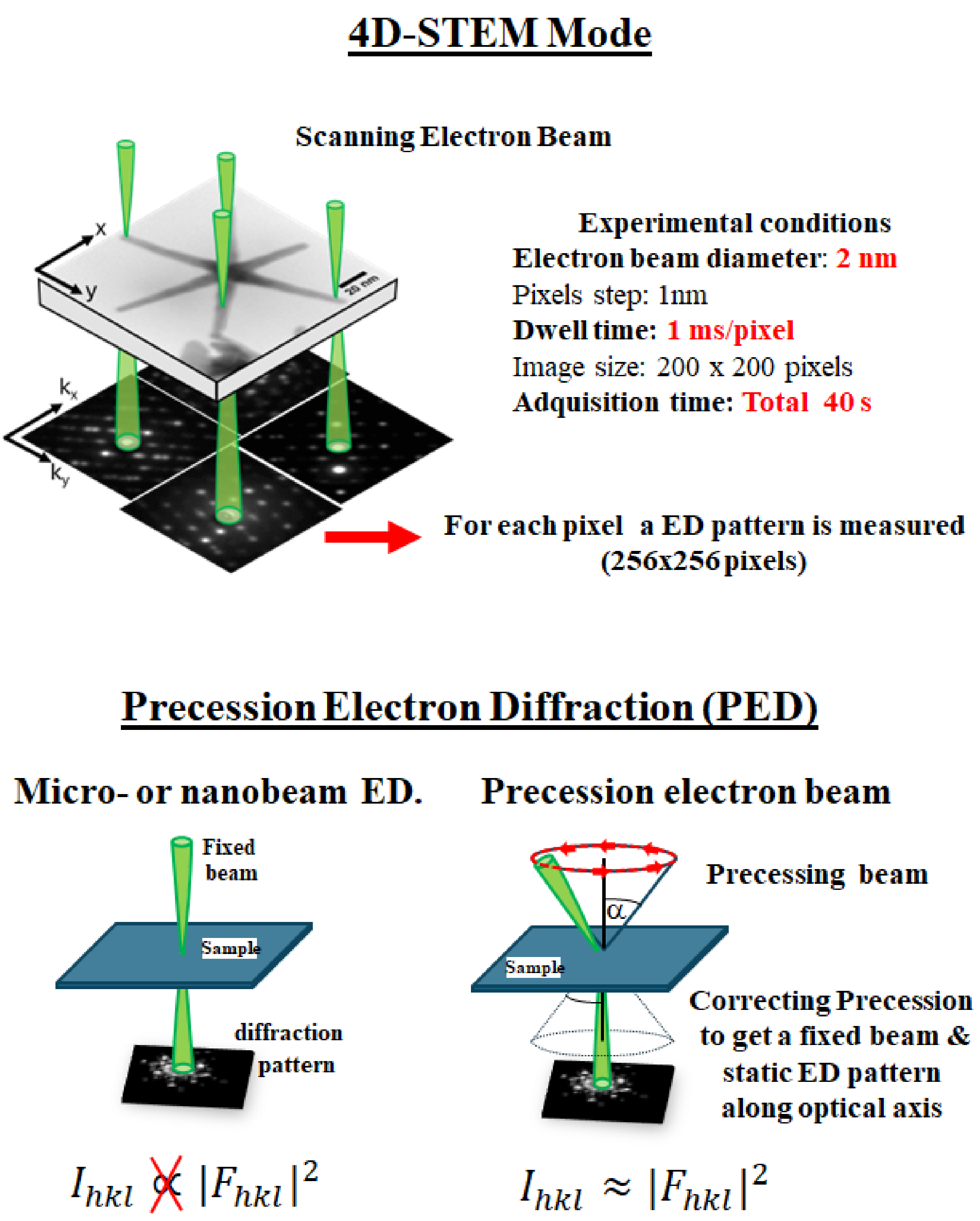

Figure S1. Scheme showing operational principles of 4D-STEM diffraction mapping (top) and comparison between fixed beam nano- or microdiffraction with precession electron diffraction (bottom).

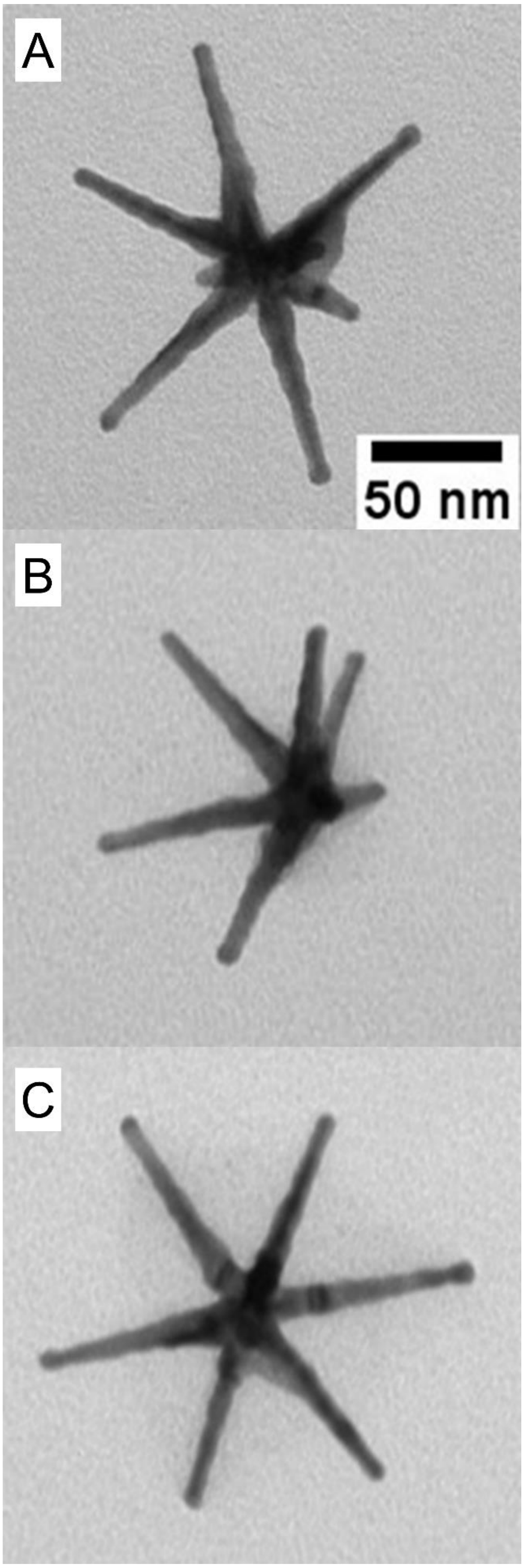

Figure S2. a-c) TEM micrographs showing a general view of 6-branched noble metal nanostar showing high aspect-ratio legs.

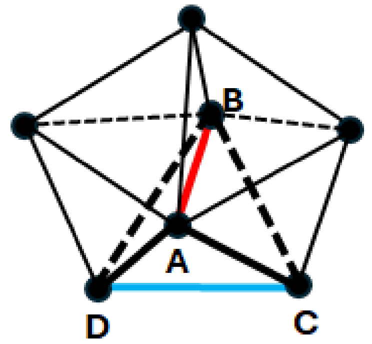

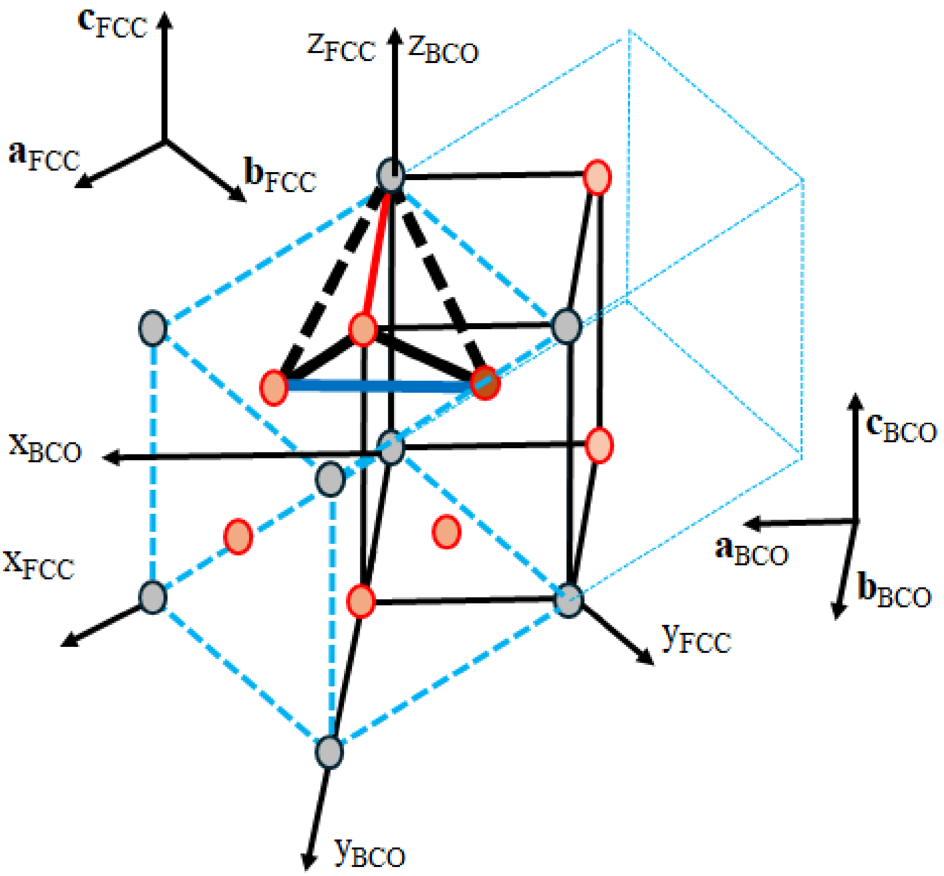

Figure S3. The atomic arrangement of decahedral particles may be described as the assembly of five tetrahedra along a common 5-fold axis. Face centered cubic (FCC) structure is the bulk atomic arrangement for noble metals, but filling space requirement induce an expansion of the DC distance to reach 72 degrees between triangles ABD and ABC. This expansion generates a body centered orthorhombic unit cell as described by Yang (16). The bottom part of the figures shows the geometrical relation between the FCC and BCO unit ells.

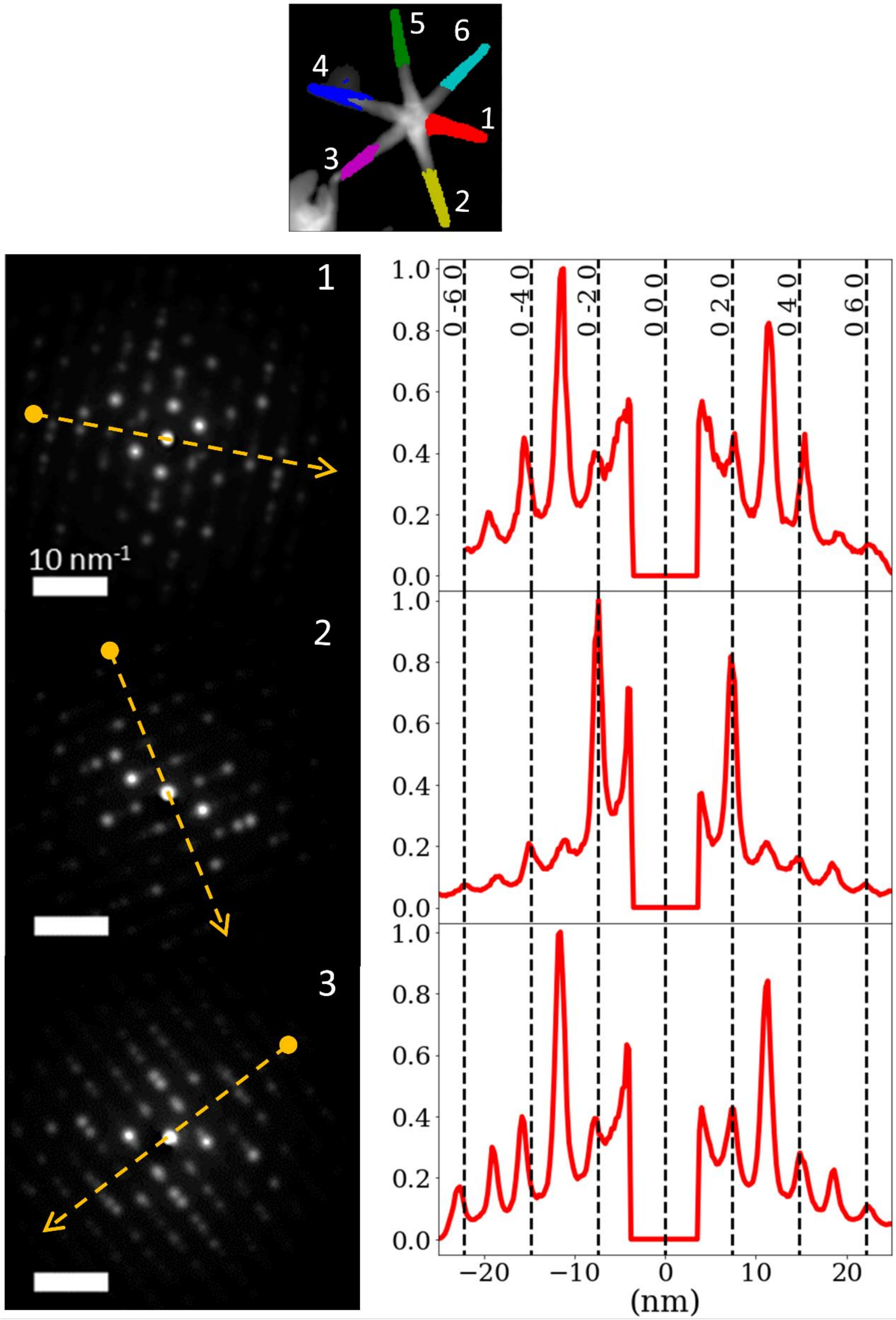

Figure S4a (continues next page). Top: Grouped pixels showing similar diffraction patterns clustered by the ML tool K-means (*17*) superimposed on the NS VDF. Below, we show, on the left side, the mean diffraction pattern calculated from region on LEG#1-6 respectively (arrows indicates leg axis on patterns and also the core to leg tip direction). On the right, we show the diffracted intensity profiles extracted along the arrows (left-to-right indicates core-tip direction). Scale bars are 10 1/nm.

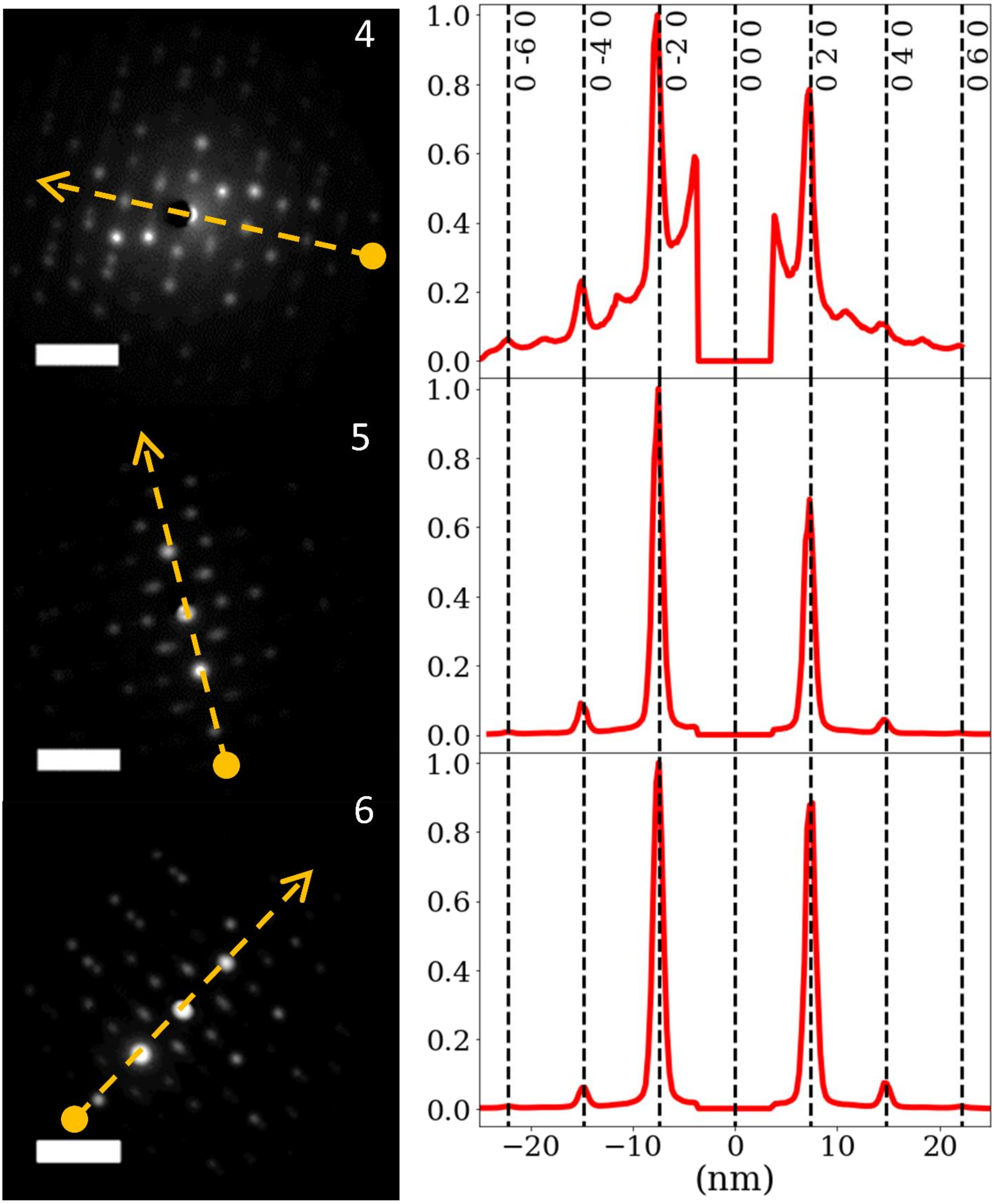

Figure S4b. Second page of Figure S4.

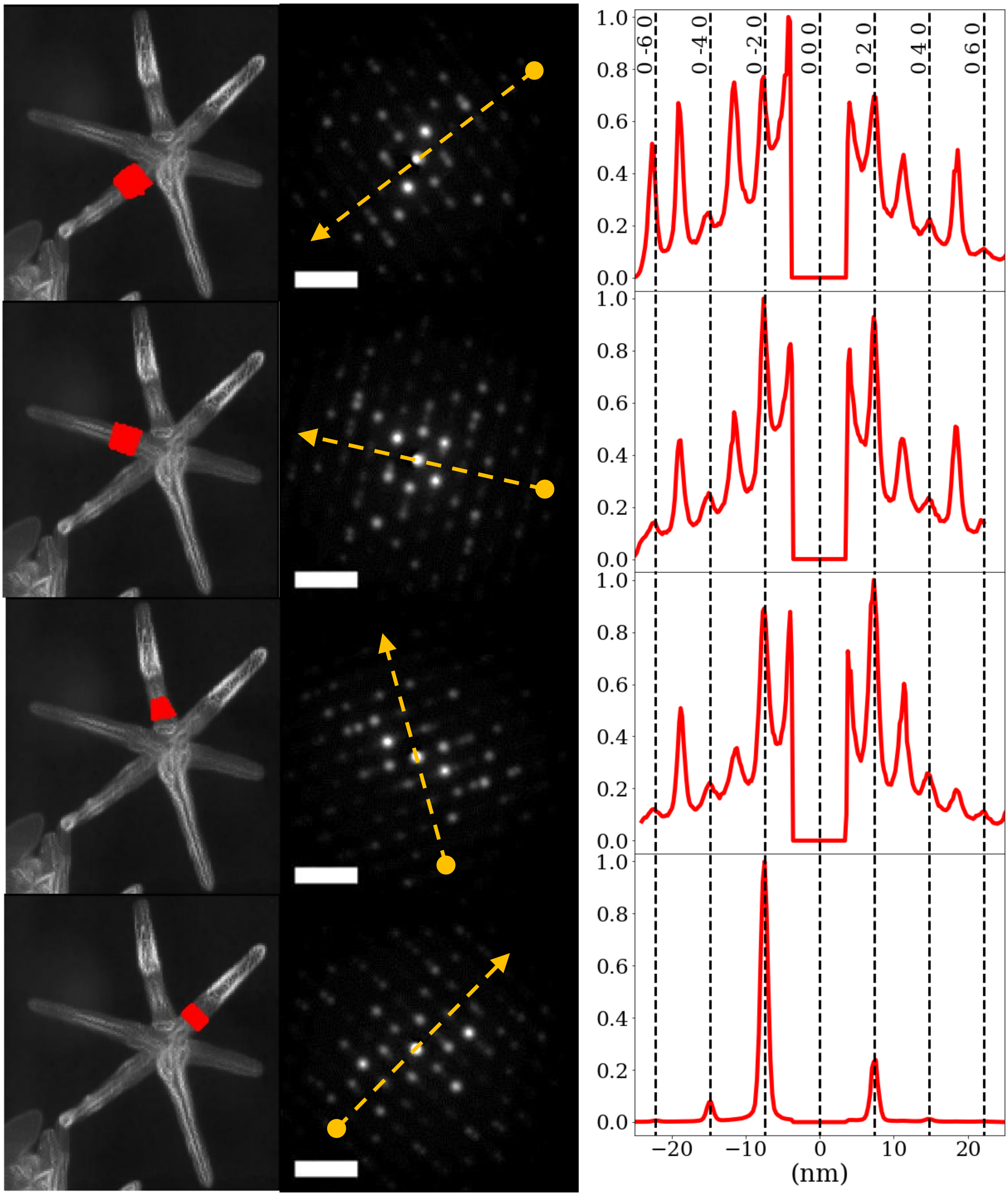

Figure S5. Mean diffraction patterns and diffracted intensity profiles (extracted along the arrows, left-to-right indicates core-tip direction). From manually selected pixel regions (leg bases) close to the NS core centre from on LEG#3-6. Scale bars are 10 1/nm.

Rough Crystal Orientation Estimation (Manual)

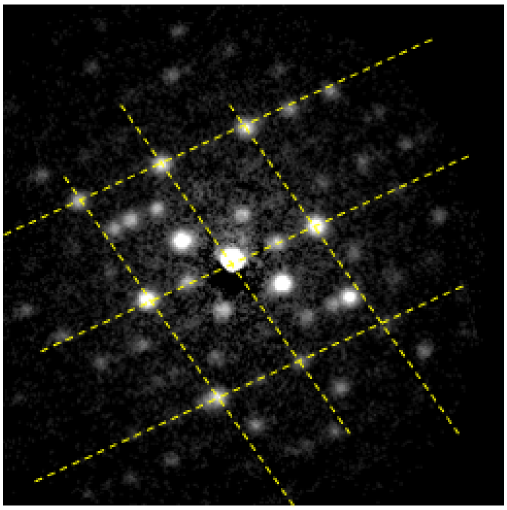

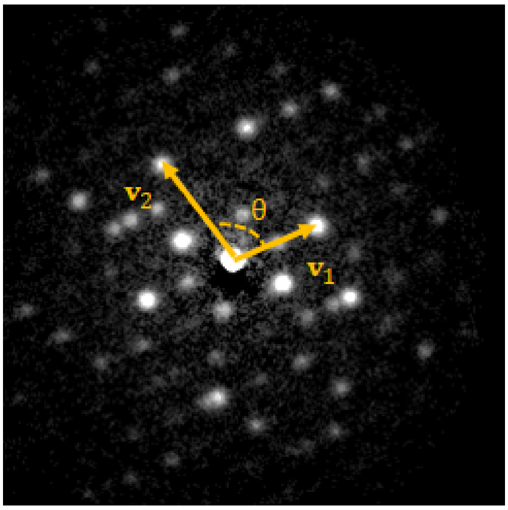

(a) Manual association of reciprocal vector lengths with interplane distance in real space

$$|\mathbf{v}_1| = \frac{1}{d_{h_1 k_1 l_1}}$$

$$|\mathbf{v}_2| = \frac{1}{d_{h_2 k_2 l_2}}$$

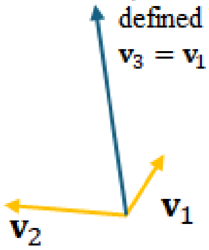

b) Normal to plane defined by $\mathbf{v}_1$ & $\mathbf{v}_2$
$\mathbf{v}_3 = \mathbf{v}_1 \times \mathbf{v}_2$

c) Rough incidente electron beam direction in relation to crystal

Fine Crystal Orientation Estimation through Quantitaive Analysis of Diffracted Intensities

(d) Measuremant of diffracted beam intensities at positions defined as:

$$\mathbf{v}_{hkl} = m\,\mathbf{v}_1 + \mathrm{n}\ \mathbf{v}_2$$

(m, n integer numbers)

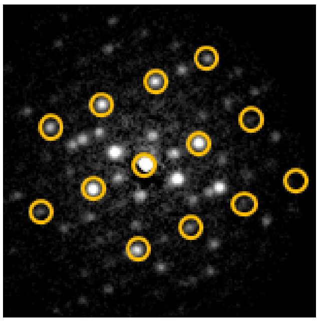

(e) Crystal Orientation refinement by applying a Rietveld-like procedure comparing experimental intensities to simulated values. A Residue is used as the metric to select the solution (or the crysal orientation) that best reproduced measured diffracted intensities in the PED pattern

Simulation and Residue

$$R_N = \frac{1}{N}\frac{\Sigma_N \left|\sqrt{I^m_{hkl}} - \sqrt{I^s_{hkl}}\right|}{\Sigma_N \sqrt{I^m_{hkl}}}$$

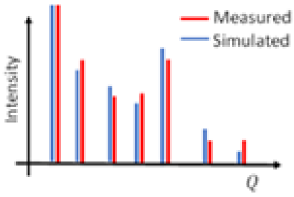

Figure S6. Top: Schematic representation of the manual identification of zone axis from a PED pattern. Bottom: procedure adopted to make a fine measurement of crystal orientation in relation to the incident electron beam, using the intensities from the diffraction spots obtained from the geometrical pattern identified by visual observation (top).

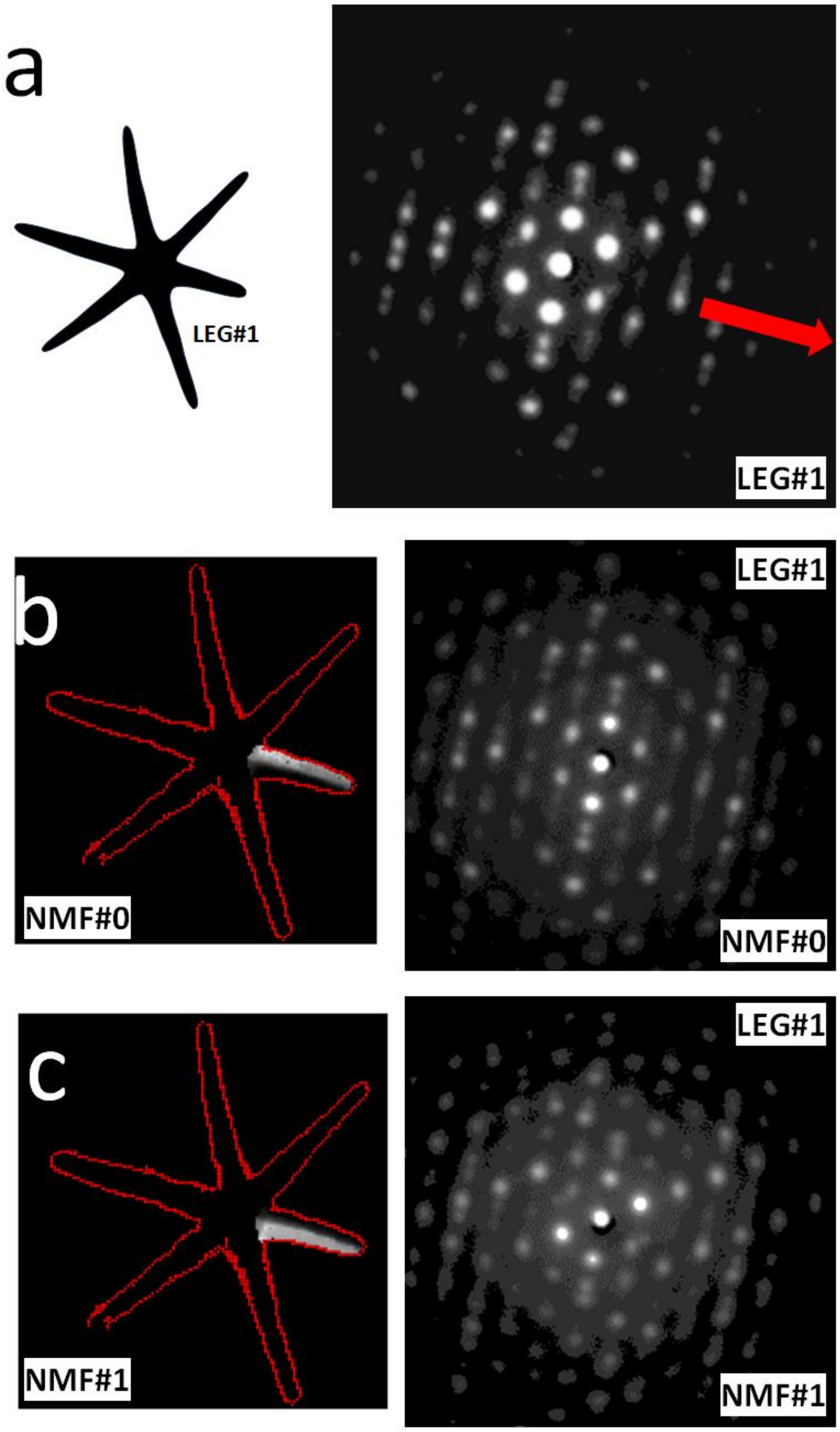

Figure S7. a) Mean diffraction pattern of LEG#1, where it is not possible to observe the peaks related to the decahedral axis $[010]_{BCO}$. Non-negative matrix factorization (NMF) has been applied to the data block formed by the LEG #1 diffraction patterns; only two components have been utilized to ensure the convergence of the method. b - c) The resulting ED patterns are very different, as well as their spatial arrangement. Notice a regular spot distribution is clearly easily recognized in component NMF#1(see details in Fig. S8).

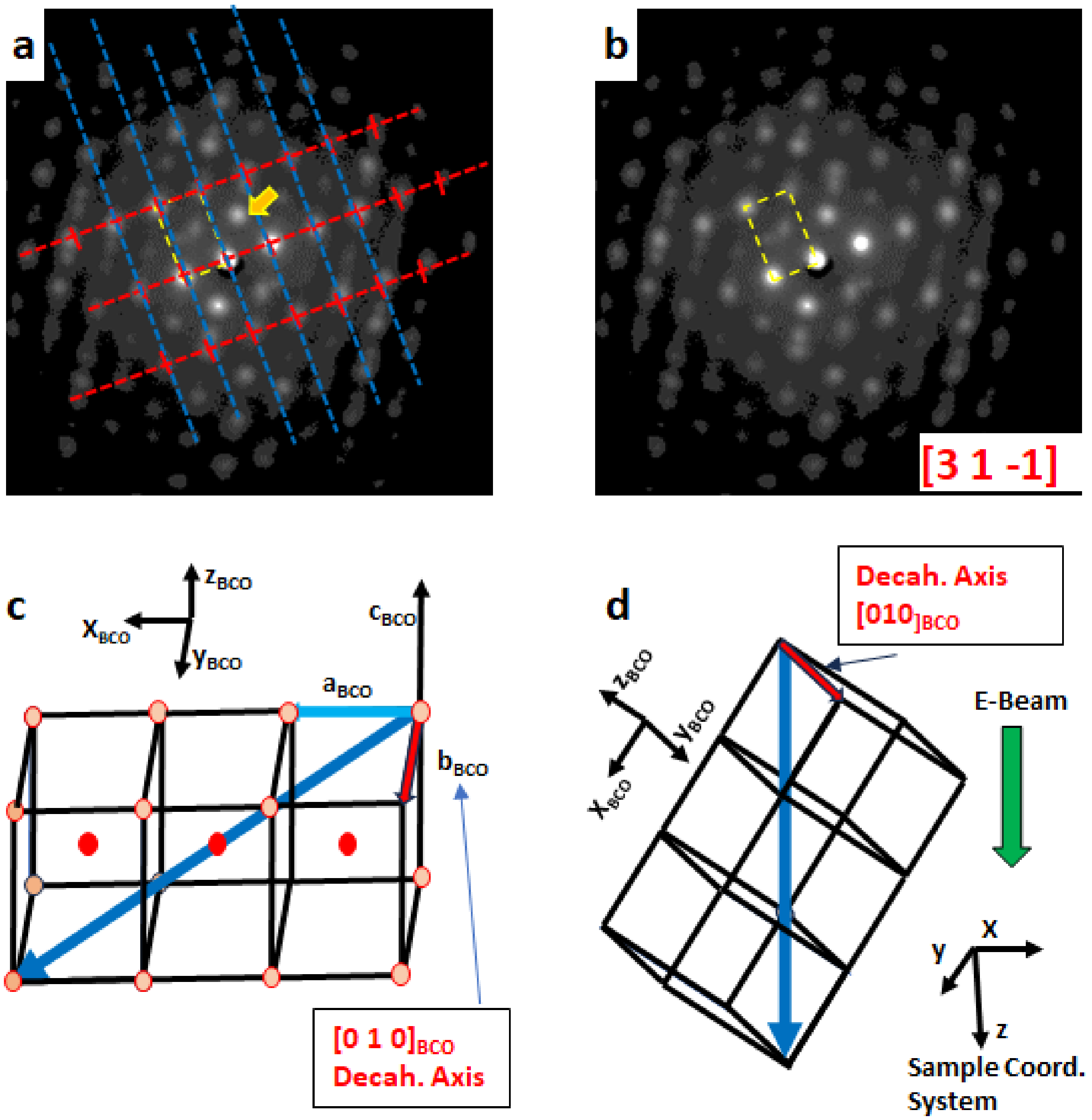

Figure S8. Analysis of ED pattern obtained as component NMF#1. a,b) A rectangular spot distribution is manually identified in this diffraction pattern, which can be indexed and a $[31\bar{1}]_{BCO}$ orientation along electron beam. c) Schematic drawing showing the identified direction in a BCO crystal; this figure also points out the expected direction of the decahedral leg ($[010]_{BCO}$). d) Deduced orientation of the leg crystal showing that the decahedral leg should point down at an elevation angle of ~17.3 degrees with the plane perpendicular to the electron beam direction. The arrow in (a) indicates a very intense diffraction spot that cannot be explained by a decahedral leg rotation of the identified BCO crystal around the leg axis to reproduce all decahedral sections of the leg. Therefore, this leg seems to be defective with a crystal in registry with the decahedral leg crystals; maybe the growth of this additional crystal may explain why this leg is slightly shorter than the other NS spikes.

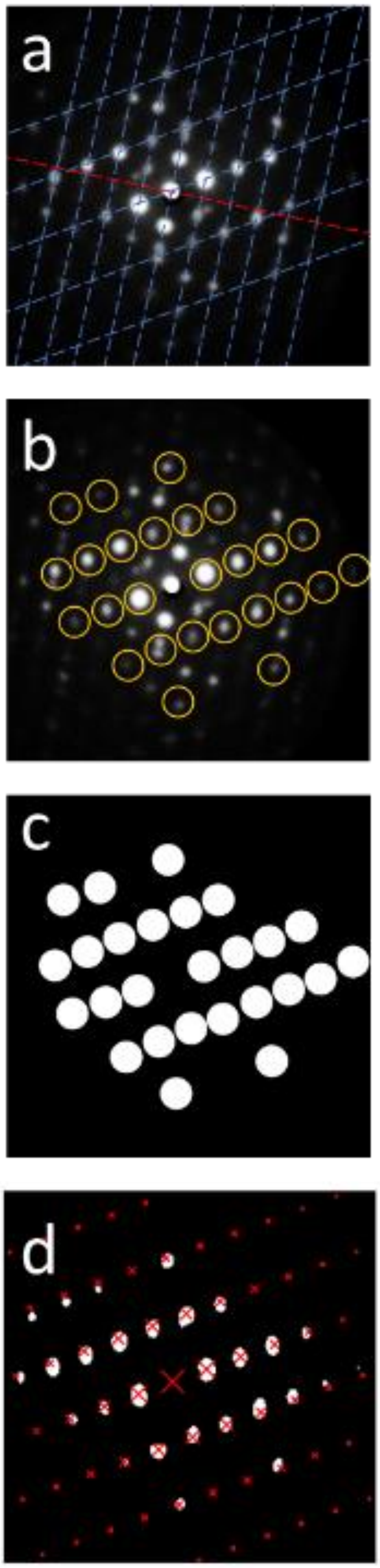

Figure S9. a) Manual identification of one of the zone axes contributing to LEG#1 after partial demixing using NMF tool. The grid represents the expected position of the peaks for a determined zone axis. b) Manual selection of the peaks for the formation of the mask in c). d) Resulting template-matching identification using Pyxem software (*17*), the crosses represent the position of the peaks for the template that best matches our data.

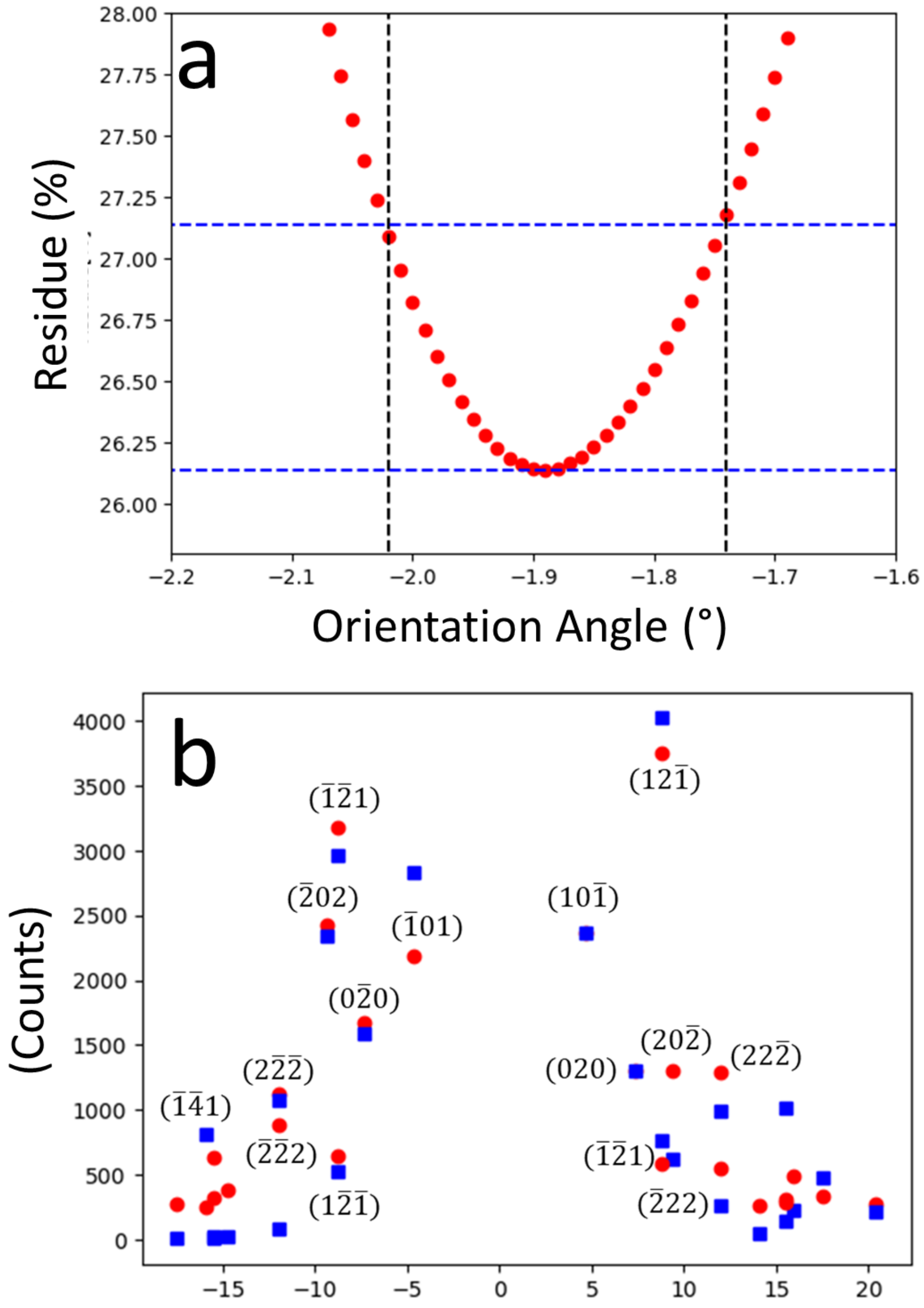

Figure S10. a) Sensitivity of the residue in relation to the elevation angle; the lines show the 1% change in residue in relation the minima. b) Comparison of the measures PED beams (circles) to the optimized values (squares).

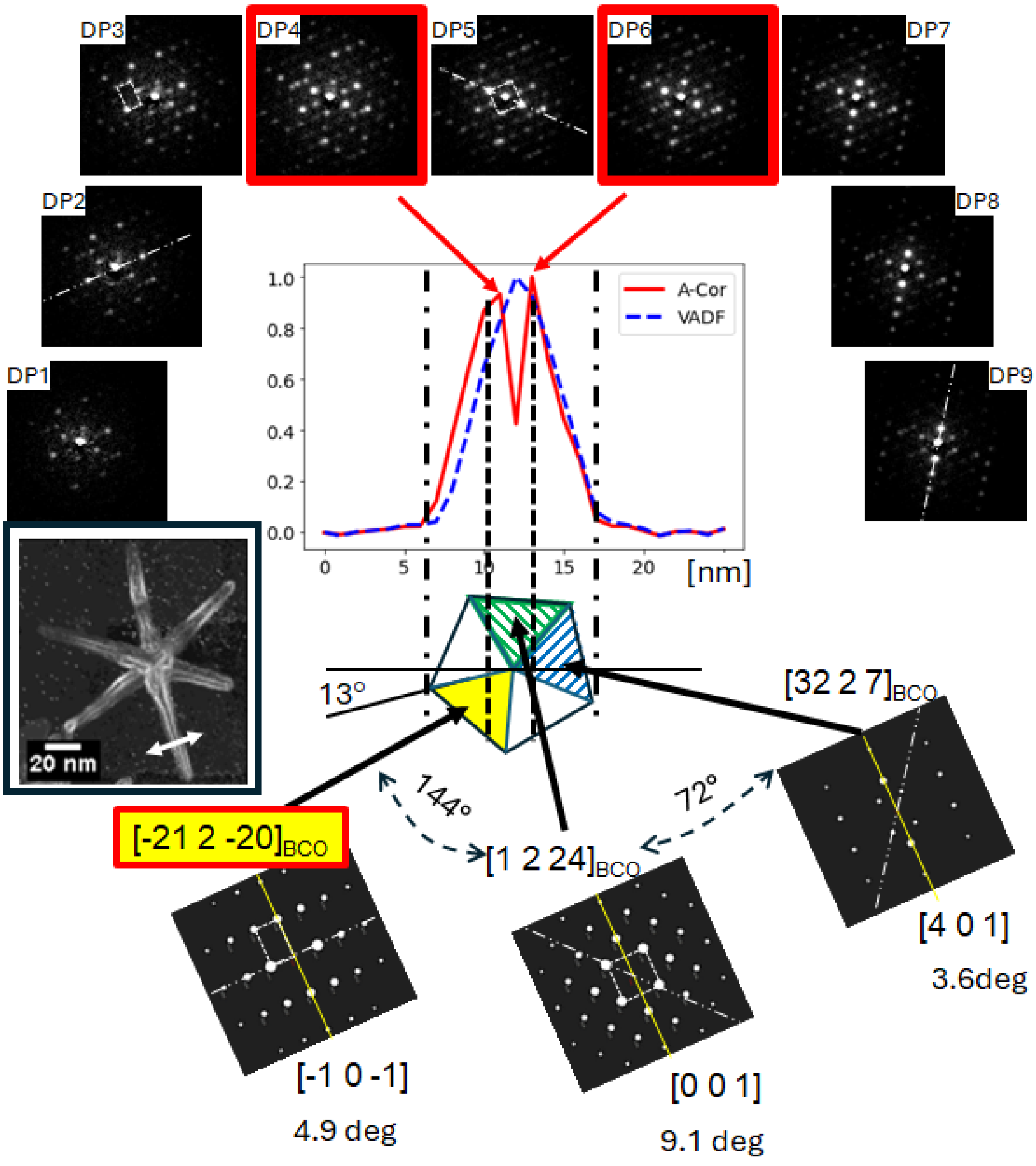

Figure S11. Analysis of diffraction patterns from individual pixels extracted along a line perpendicular to the tip of Leg#2 (see inset at the left of the figure). Top: individual diffraction patterns, note the clear changes of spots distribution from left to right; dashed lines in DP2, DP5 and DP9 provide a guide to identify the alignment direction of high intensity spots. Center: profiles of VADF and anti-correlation image intensity across Leg#2 at the same position than the DPs. Bottom: the orientation of this leg was measured from the region colored yellow at the lower left sector of the pentagonal schema (identified zone axis [-21, 2, -20]$_{BCO}$). If we rotate this crystal by 144 degrees clockwise (two 72 degrees rotation), the new zone axis is [1,2,24]$_{BCO}$; further 72 degrees rotation leaves the crystal at the [32,2,7]$_{BCO}$ zone axis. The bottom of the figure show electron diffraction patterns simulated using kinematical theory (ReciPro software (18)), and along low index zone axes close to the experimentally derived ones ([-1,0, -1]$_{BCO}$, [0,0,1]$_{BCO}$ and [4,0,1]$_{BCO}$). Their angular deviation from the directions identified from experiments at the top of the figure is indicated. The solid yellow line in simulated pattern indicates the [0,1,0,]$_{BCO}$ direction, which should be the decahedral structure axis, and dashed white lines indicate the line of high intensity spots in agreement with experimental measurements from individual pixels DPs. These simulations help to understand diffraction spots from the individual pixels, for example, the rectangular (square) geometrical distribution of spots in the simulated [-1,0, -1]$_{BCO}$ ([0,0,1]$_{BCO}$) patterns is clearly observed in DP3 (DP5). Finally, high intensity spots in DP7-DP9 are aligned close to a vertical line whose angle with the [020]$_{BCO}$ direction is ~35 degrees, as can be measured from the simulated patter along [4,0,1]$_{BCO}$ pattern. The very good agreement between experimental data and simulations provides a solid basis for the interpretation of the legs as decahedral wires.

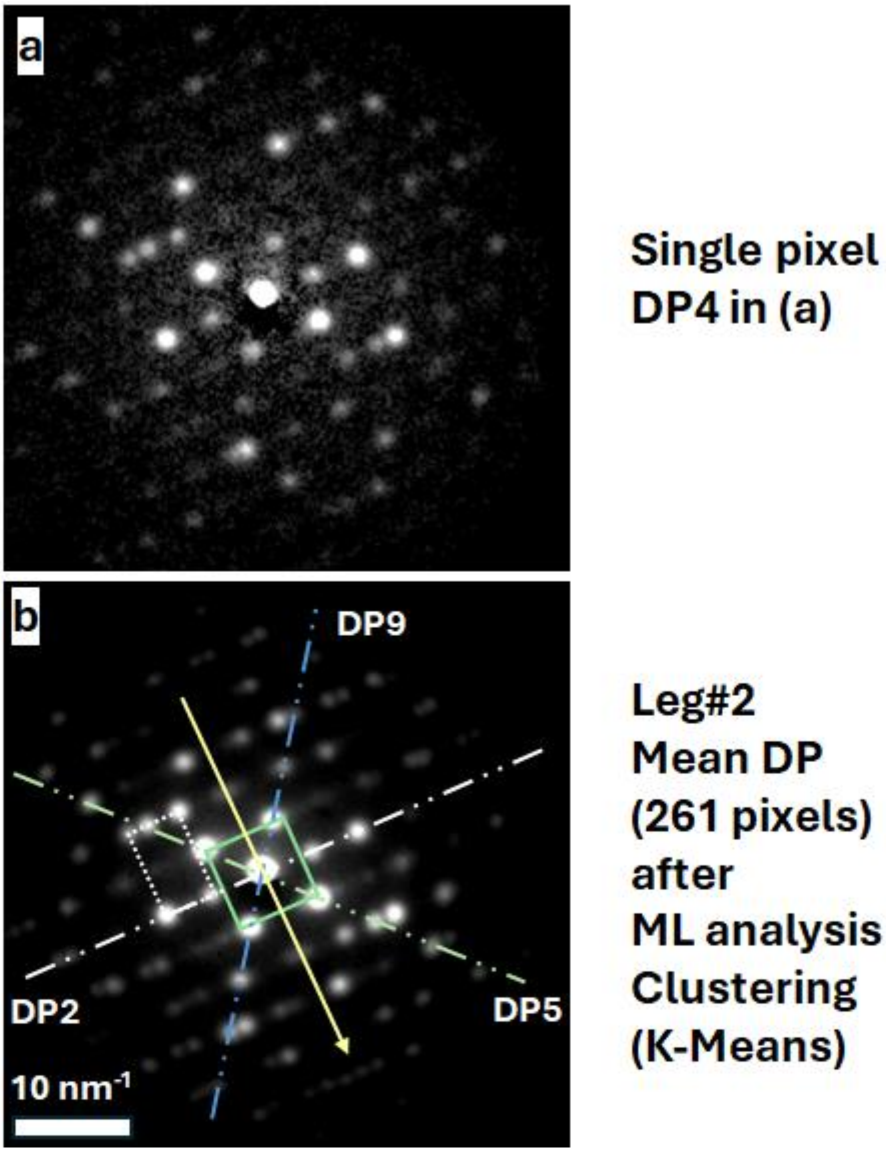

Figure S12. Comparison between the individual pixel diffraction pattern marked DP4 in (a) and the average diffraction pattern generated by ML tools in (K-Means for the tip of leg#2). The interpretation of this kind of patterns is rather complex because it is generated from the superposition of 5 different crystals from the decahedral leg. Dashed white lines indicate the direction of high intensity spots in (DP2, DP5 and DP9); the rectangular square distribution of spots as well as their orientation can be easily correlated with simulation in Fig. S11

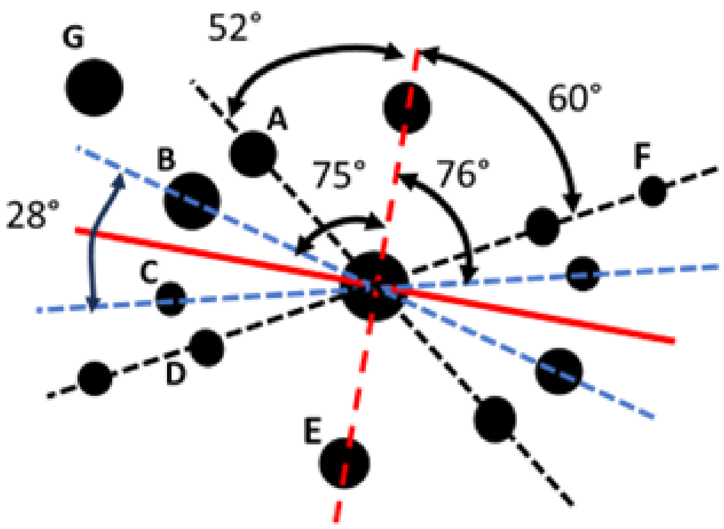

Real Space Distances

SPOTG	d_{hkl} [Å] (±0.05)
A	2.23
B	1.94
C	1.93
D	2.23
E	2.23
F	1.42
G	1.17

Figure S13. A schematic draw of the mean diffraction pattern from NS central region derived from diffraction spots from the mean diffraction pattern from the core (4D-STEM pixels at a radial distance < 10 nm). Measured values of angles between diffraction peaks and the correspondent real space distances are shown.

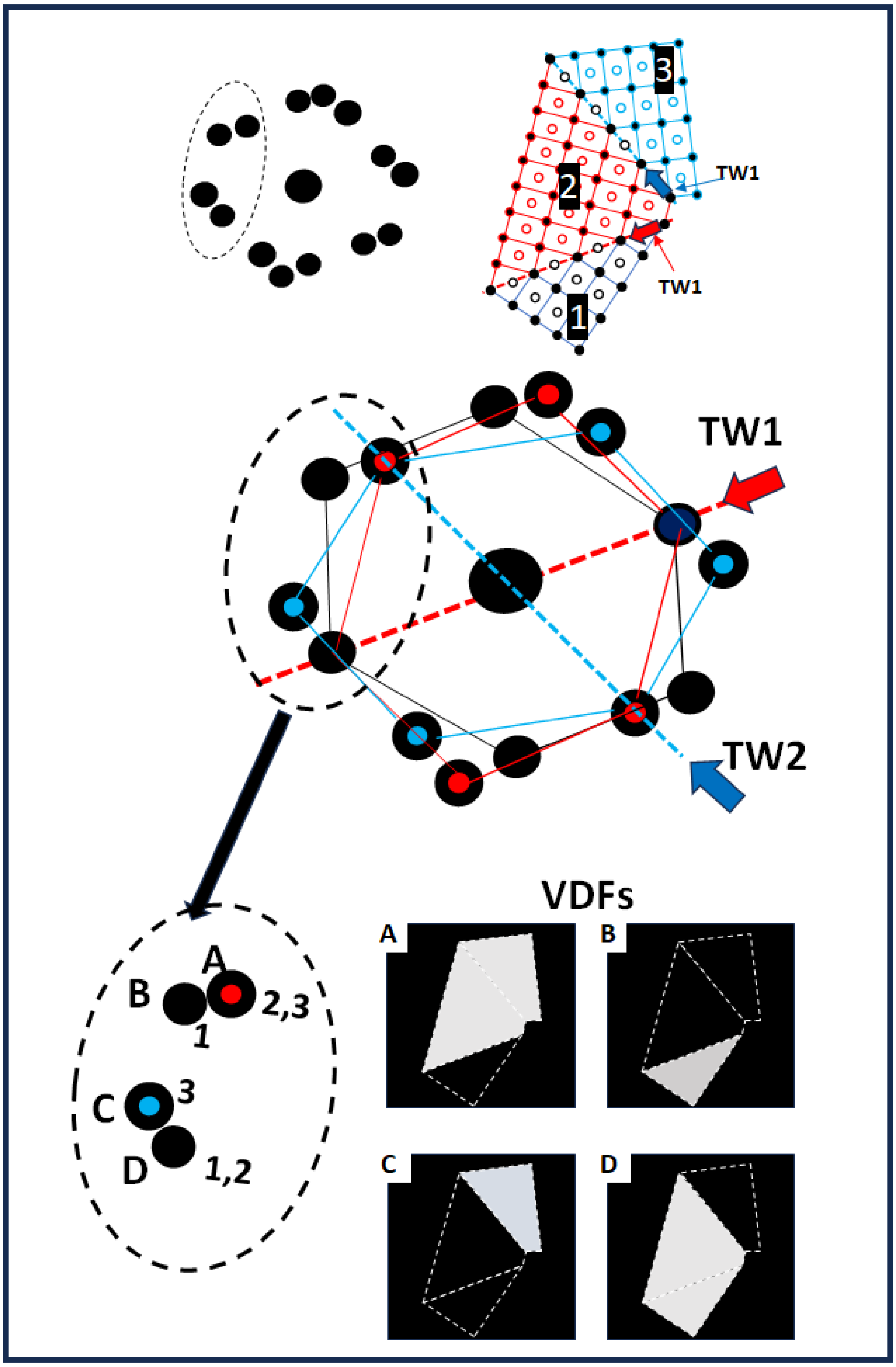

Figure S14. A schematic illustration showing the expected SAED pattern generated from face-centred-cubic (FCC) lattice oriented along [110] axis including three crystals (1-3) connected by two mirror twins (TW1 and TW2). A simple analysis of the diffraction pattern allows the identification of twin positions and the correlation between crystal number (bottom part of the figure) and diffracted spot (see Figures 9.3 and 9.4 from pages 524 and 525 from book by Graef (19)). For example, a VDF generated by spot A should show crystal #2 and #3, while a VDF from spot C must only show crystal #3. Then, we can use a series of VDF images displayed in Fig. 3 exclude the twinned FCC crystal occurrence.

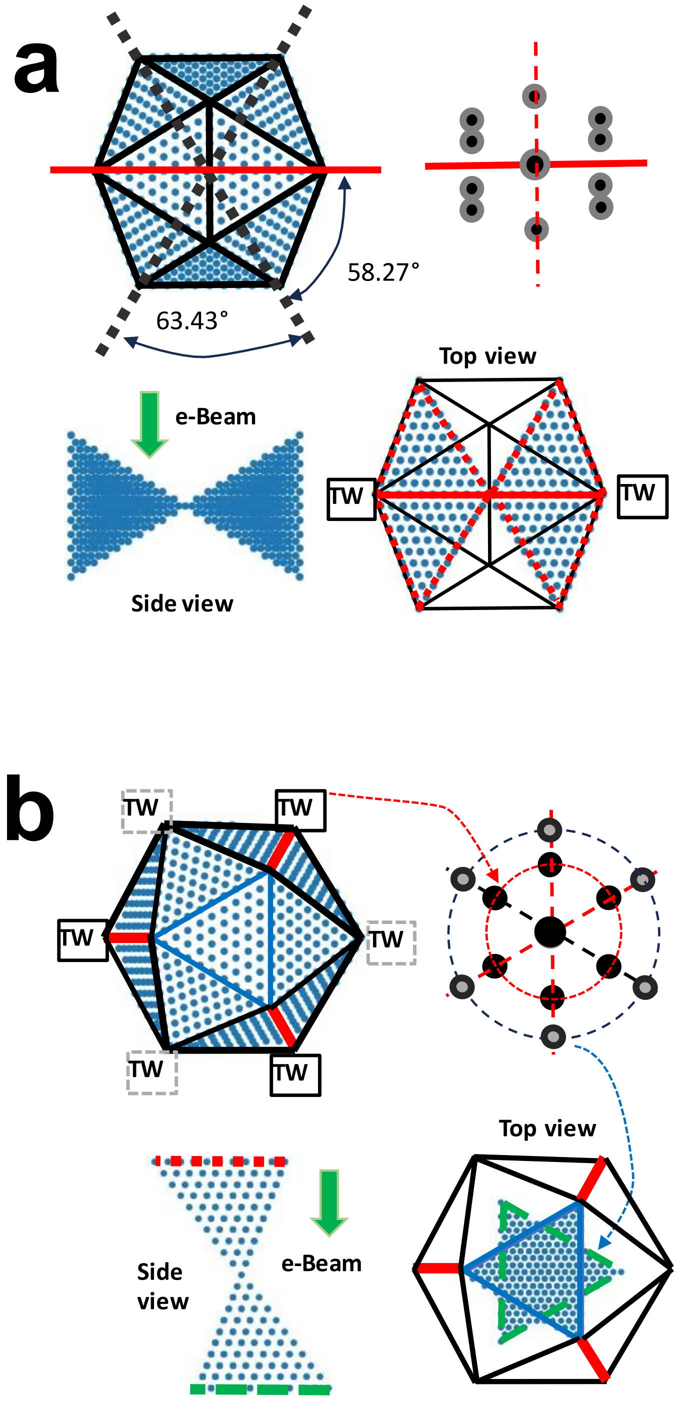

Figure S15. Schematic drawing of the structural aspects associated to ICO particles oriented along 2-fold and 3-fold axes including projection images (top- and side-views) and diffraction patterns. a) Along the 2-fold axis, the ICO ED appears to be a twinned crystal, because groups 2 tetrahedra at each side of the ICO are oriented on zone axis. Along the 3-fold axis (shown in (b)), the diffraction pattern shows an apparent 6-fold symmetry where two different families of spots (or arising from different crystal) are perfectly aligned to each other. The inner ED spots are associated to 6 twins that become parallel to the electron beam; the larger circle of diffraction spots (or shorter real space distances) is associated to two tetrahedra located at the centre of the ICO image whose $(111)_{RHO}$ facets are perpendicular to the incident direction.

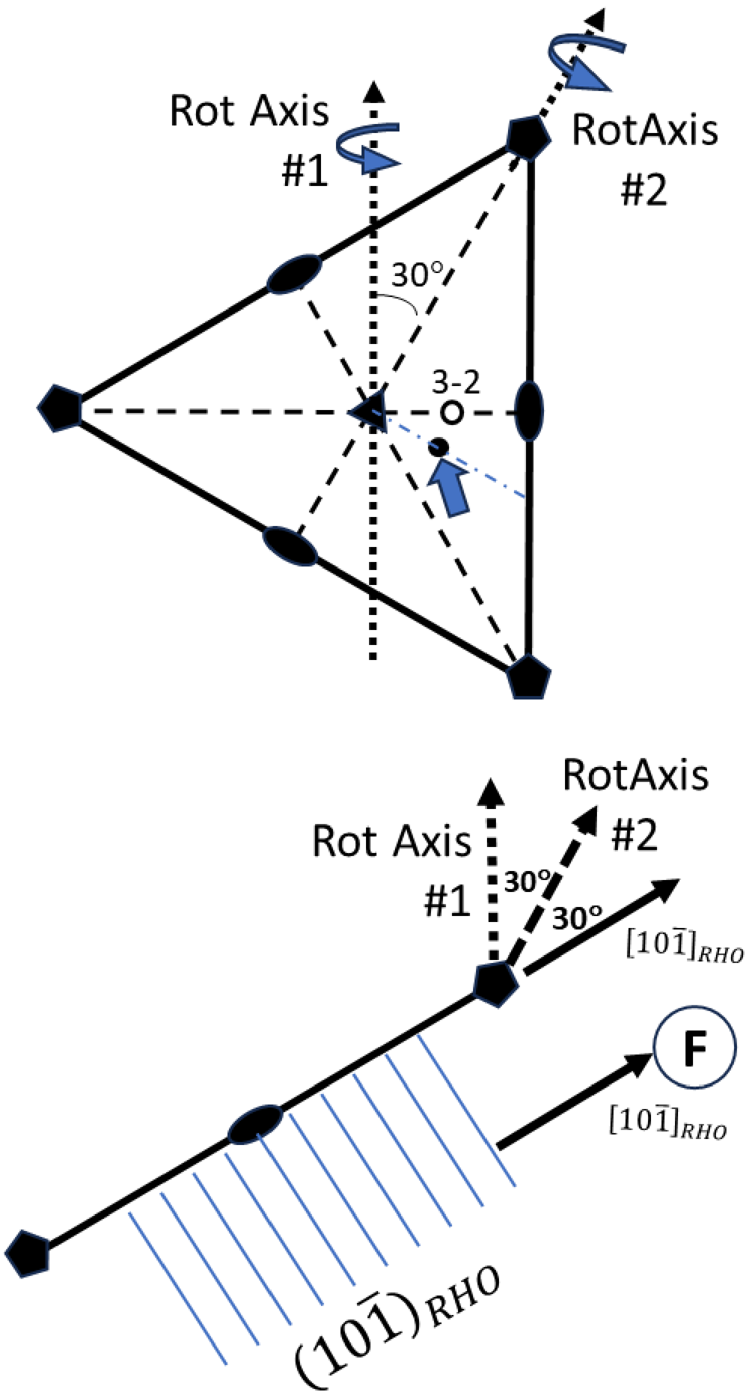

Figure S16 A schematic drawing indicating high symmetry axes of the icosahedra by observing a regular triangular facet. Axes with 5-fold symmetry are located at facet corners, 3-fold axes at the triangle centre, and 2-fold axes are located at the centre of edges. Just by rotating the particle around the vector noted Axis#1, we can move between the three types of axes. The intermediate direction between a 2-fold and 3-fold axis (noted 32, 10.5 degrees from both axes). To preserve a good orientation of planes generating the diffraction spot marked F, the ICO must be rotated along their normal (or the reciprocal vector $[101]_{RHO}$, see lower part of the figure). This axis is located at 60 degrees from Axis#1, and a rotation around this axis will strongly diminish the influence of the 2-fold axis in the final ICO diffraction pattern, in contrast with experiments. We have explored the crystallographic characteristic of an ICO particle rotated around an axis (Axis#2) at an intermediate position between Axis#1 and $[101]_{RHO}$. This orientation (arrowed in the drawing) will be called ICO32D, to note a deviation for the intermediate position between ICO2 and ICO3.

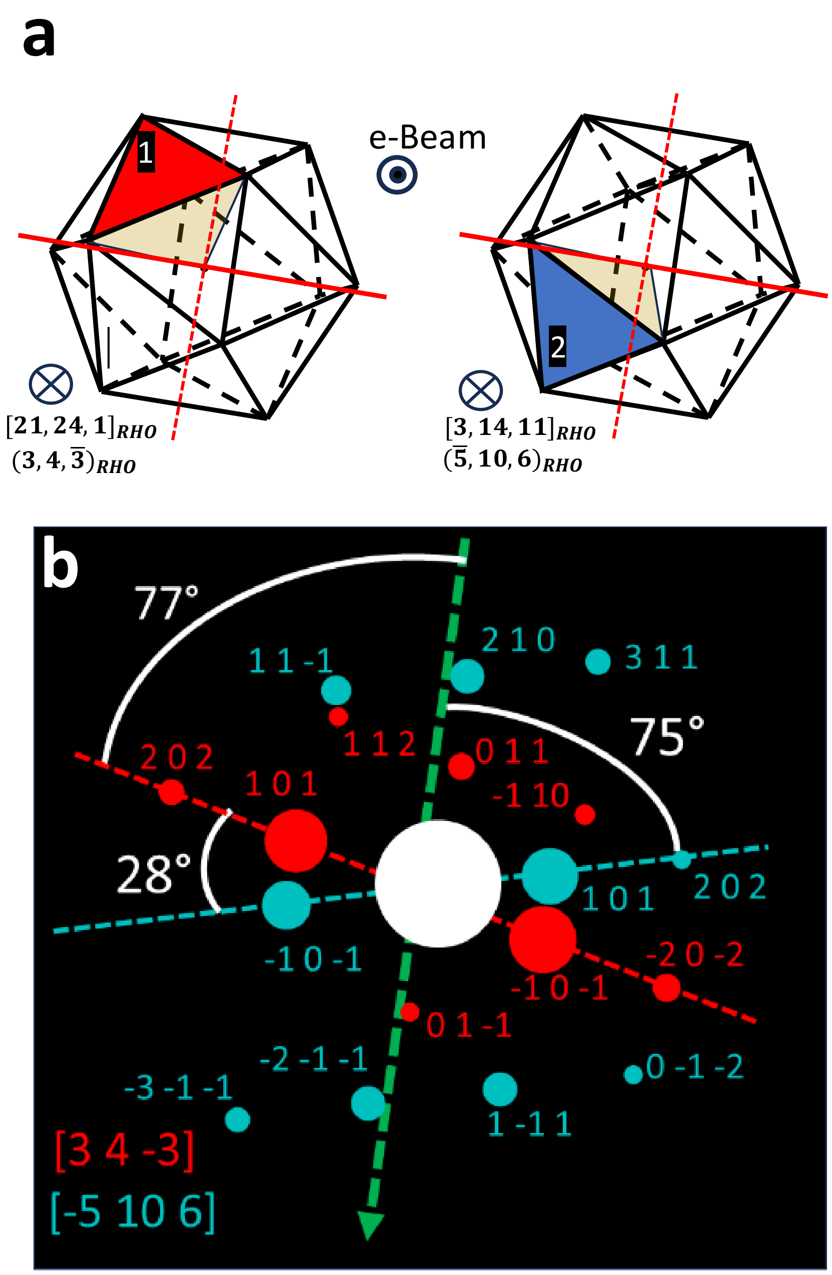

Figure S17. a) The expected orientation of the core in between the ICO23D direction which implies that the tetrahedron marked #1 is oriented along the [21 24 1]$_{RHO}$ direction in real space ([3 4 -3]$_{RHO}$ in reciprocal space. Simultaneously, tetrahedron #2 is oriented along the [3 14 11]$_{RHO}$ direction in real space ([-5 10 6]$_{RHO}$ in reciprocal space). Kinematical simulation of the rhombohedral lattice oriented along [3 4 -3]$_{RHO}$ and [-5 10 6]$_{RHO}$ zone axis obtained with ReciPro software (*18*). Notice that peaks along the [101]$_{RHO}$ directions are dominant in both zone axis, with an angle of ~75° with the *y* direction (defined as the normal to twin plane perfectly oriented when ICO is oriented along ICO32 direction). The intensities of the [3 4 -3$_{RHO}$ are higher than the ones for the [-5 10 6]$_{RHO}$, as predicted for the model orientation (see Fig. S16) and in agreement with the experiment (see Figure 3 and S13).

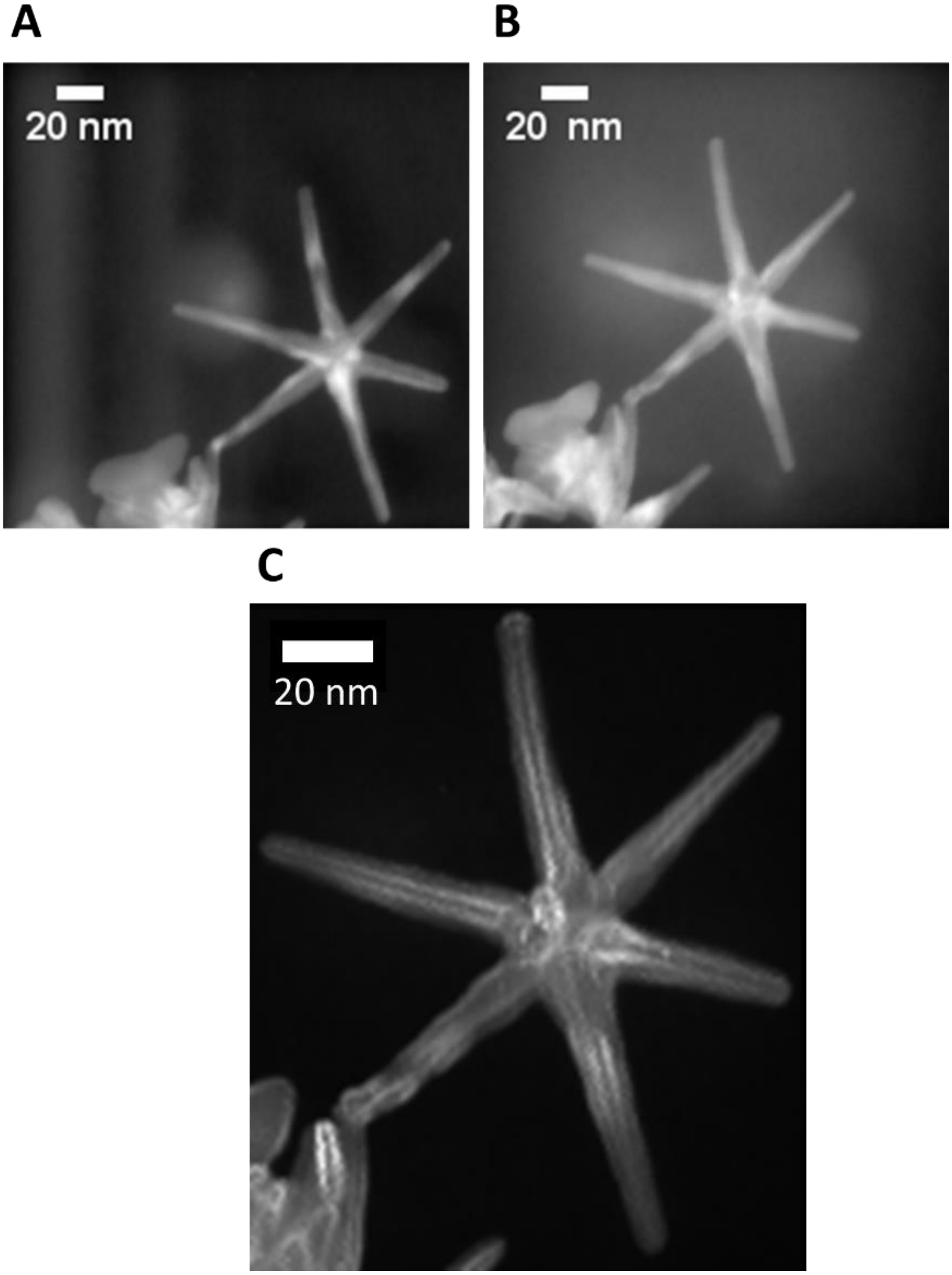

Figure S18. VADF of the nanostar: a) 0 degrees and b) 10 degrees dataset. Notice that the image in (b) shows an increased background intensity due to a-C contamination. c) Anti-correlation image of the nanostar from the 10 degrees data set, where the polycrystalline nature of the legs is clearly evident (bright lines along legs indicate twin positions), implying that the decahedral atomic arrangement of the thin high-aspect ratio legs has been preserved after data acquisition.

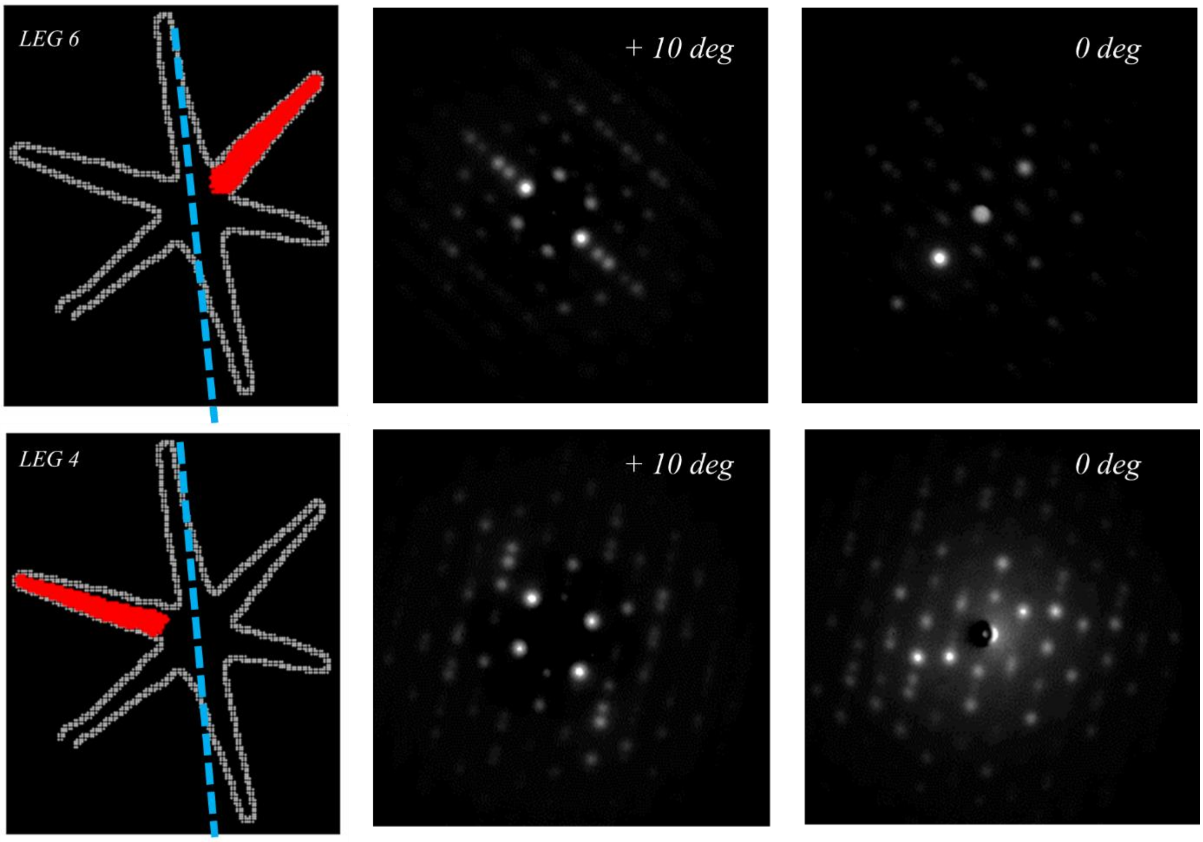

Figure S19: Legs 4 (upper) and 6 (lower) after a sample rotation of +10 degrees using the TEM goniometer (nominal value). The +10 degrees dataset has been analysed with the same procedure previously described. A clustering procedure has been capable of differentiating and isolating each leg (the left image shows the clustered regions for legs 4 and 6). The mean diffraction patterns of each leg changes significatively after rotation.

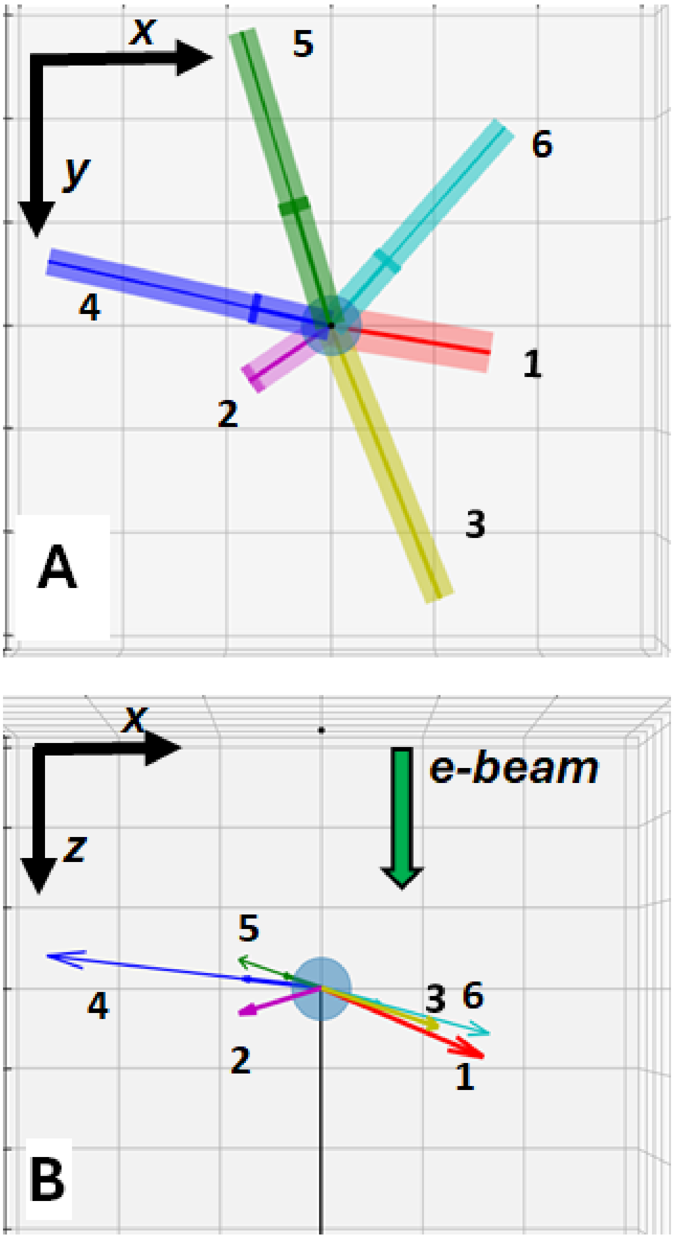

Figure S20: Nanostar 3D reconstruction for the tilted data (10 degrees). Legs#4 and 6 orientations have been measured with the PED intensity analysis, Legs#2, #3, #5 have utilized only the template-matching analysis and leg 1 has utilized only manual indexation. The angular resolution of Legs#1, 2, 3, 5 is significantly affected in relation to Leg# 4 & 6, but the legs are still restricted to a tilted plane, as expected for a planar leg distribution.

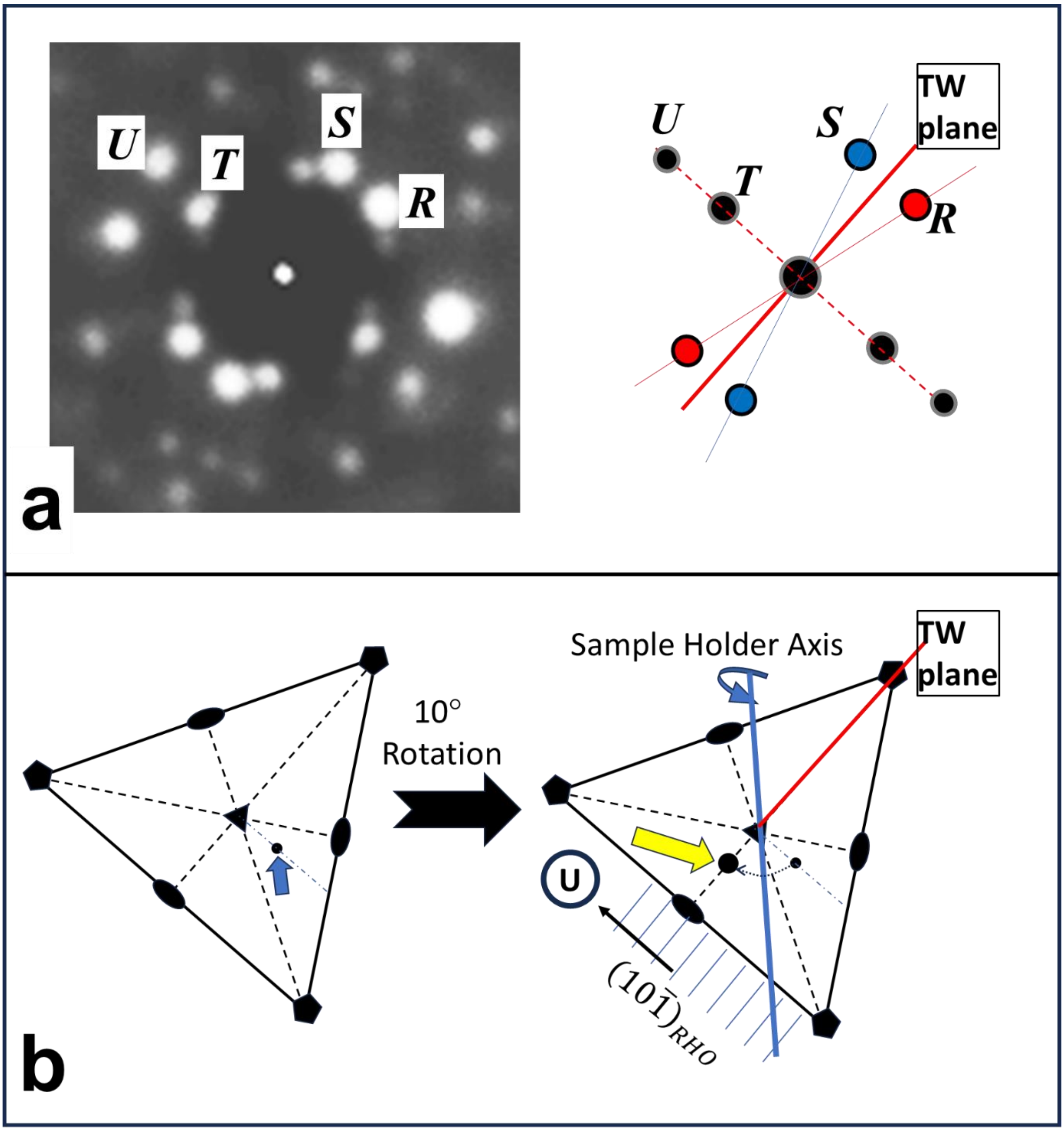

Figure S21. a) Measured PED pattern for the core of the NS for the 10 degrees dataset. b) Orientation of the NS core in relation to the ICO symmetry sites for the 0 degrees (left) and 10 degrees (right) datasets. The circle represents the estimated orientation (arrowed), ellipses the 2-fold symmetry sites, triangles the 3-fold sites and pentagon the 5-fold sites.

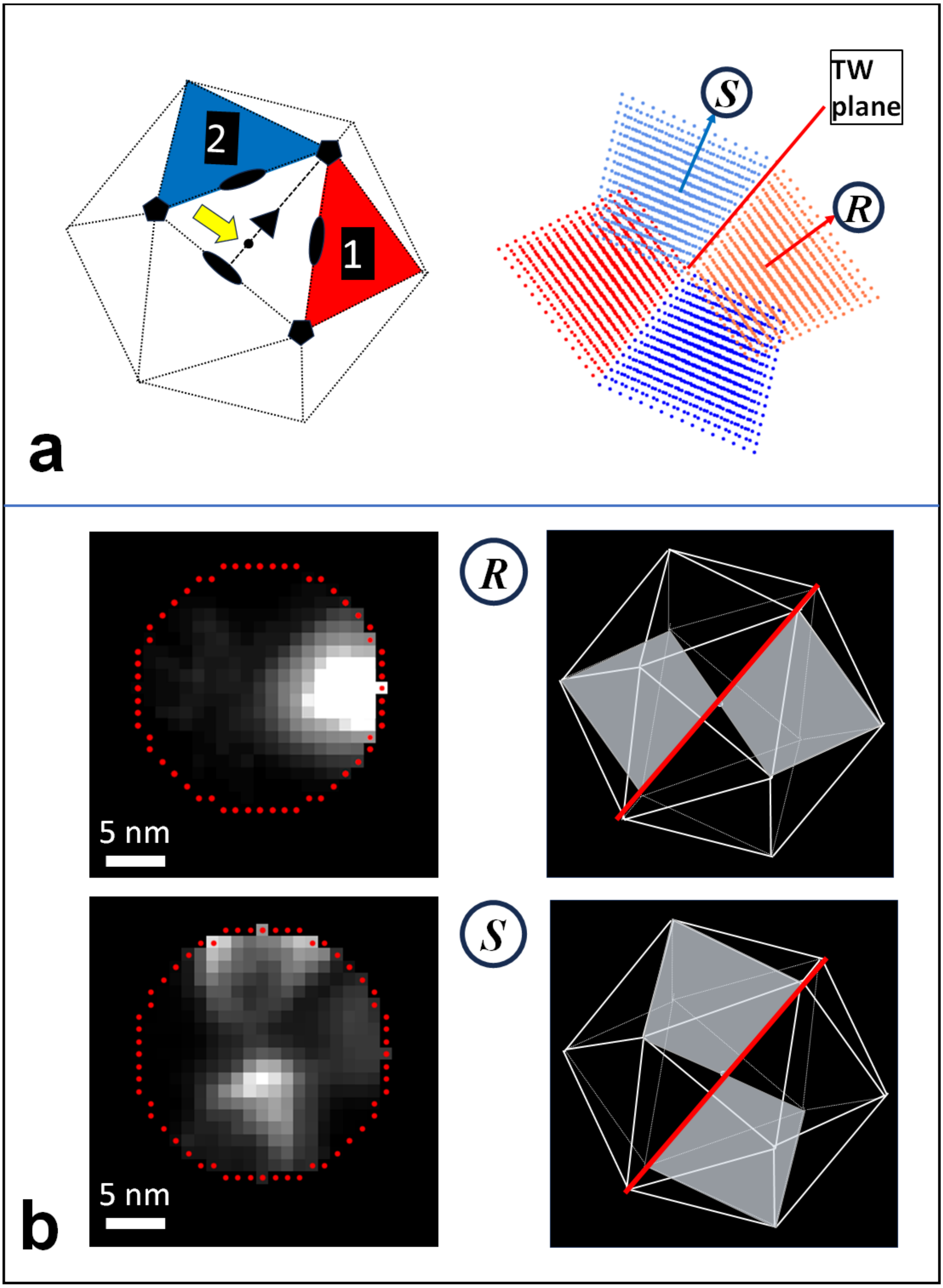

Figure S22. A) deduced orientation of the NS icosahedral core after the +10 degrees rotation; in this configuration two pairs of tetrahedra show atomic planes oriented along the electron beam direction. These planes should generate diffraction spots (*S* and *R*) of identical intensity, in full agreement with experimental data. b) Comparison of experimental and expected VDF images for diffracted beam *S* and *R*, that again show an excellent agreement.

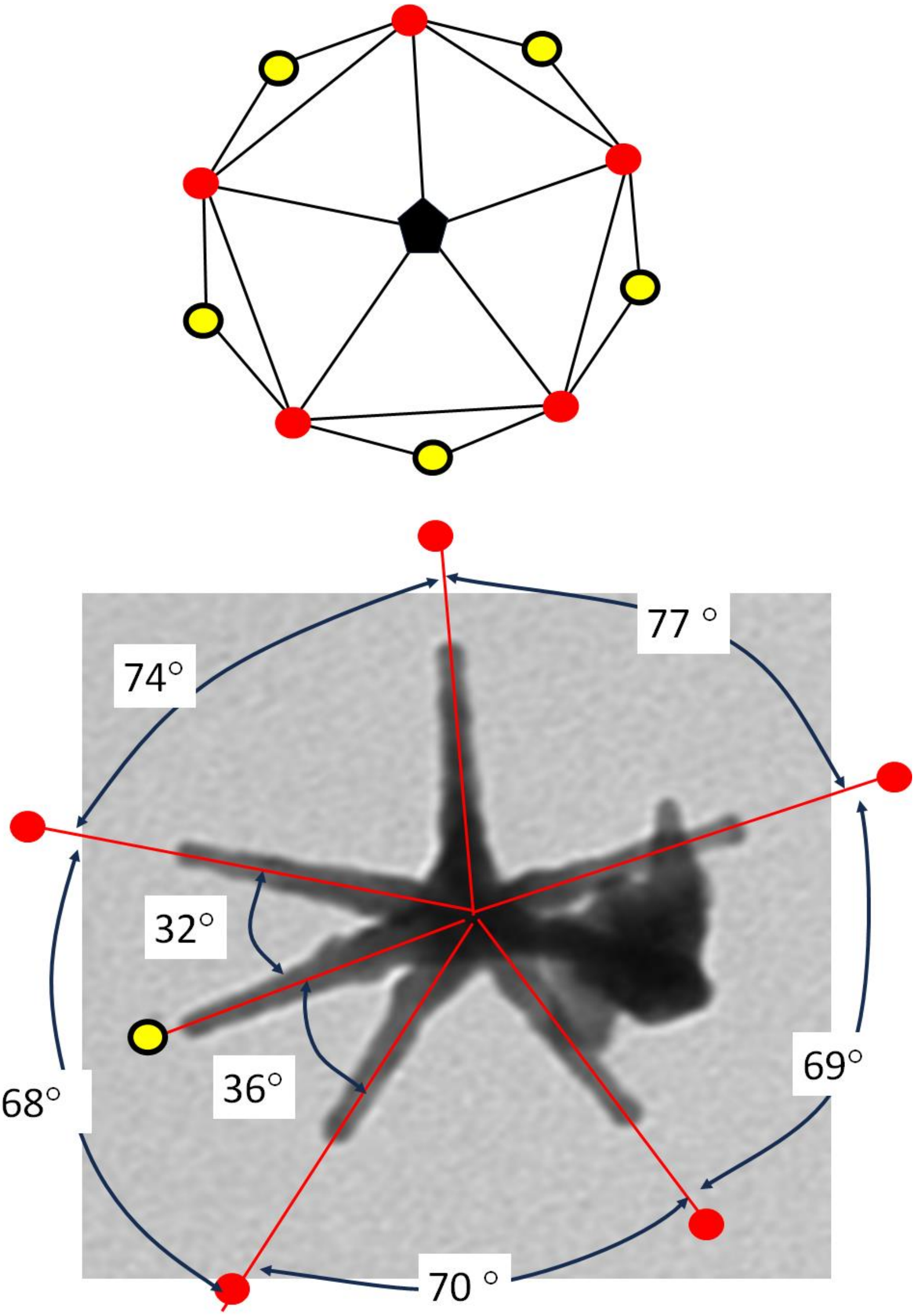

Figure S23. Image of a NS showing an ensemble of legs displaying a 5-fold symmetry distribution (marked with red disks). A 6th leg (marked with a yellow disk) is located close to the bisecting angle between legs (left lower region); this 6th leg must be growing from an icosahedral apex located along the second stacked decahedron of the icosahedral NS core (see schematic drawing included at the top).

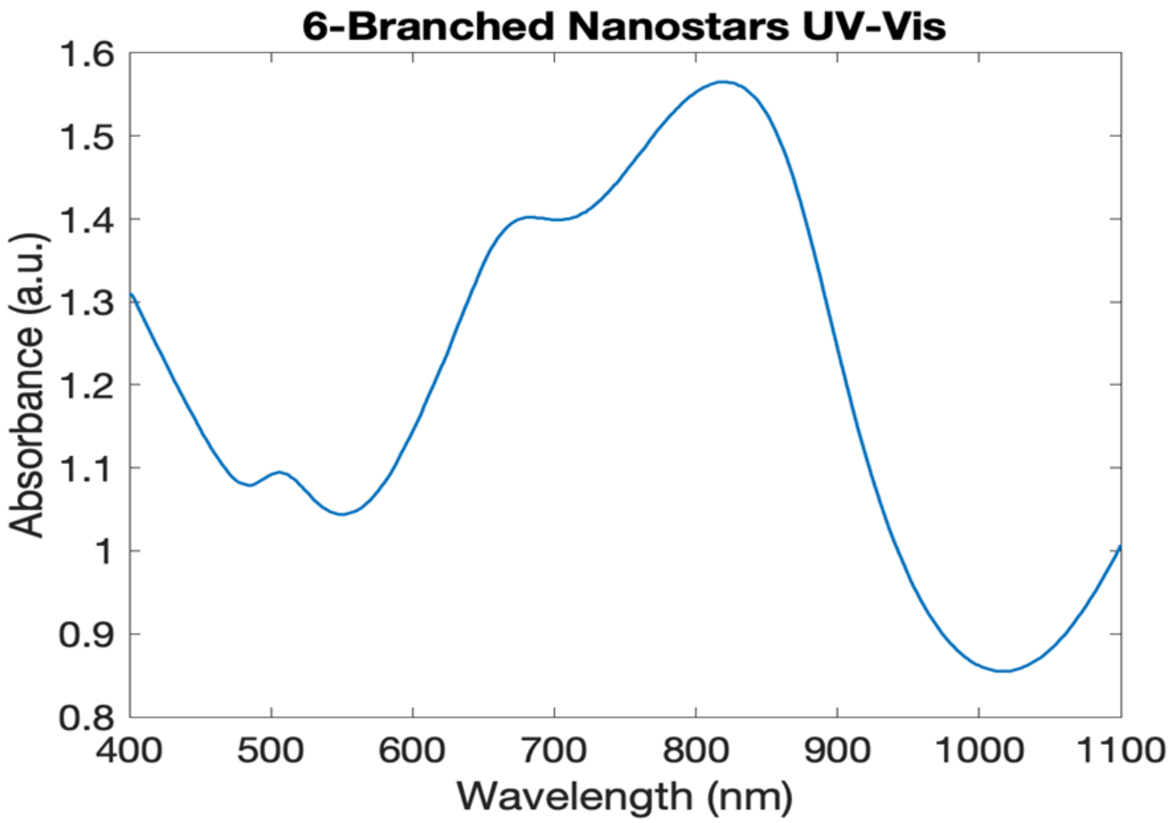

Figure S24. a) UV-Vis-NIR spectra of the 6-legs noble metal NS sample. The resulting UV-Vis-NIR spectra is characterized by prominent LSPR modes at 670 nm and 830 nm in this wavelength range.

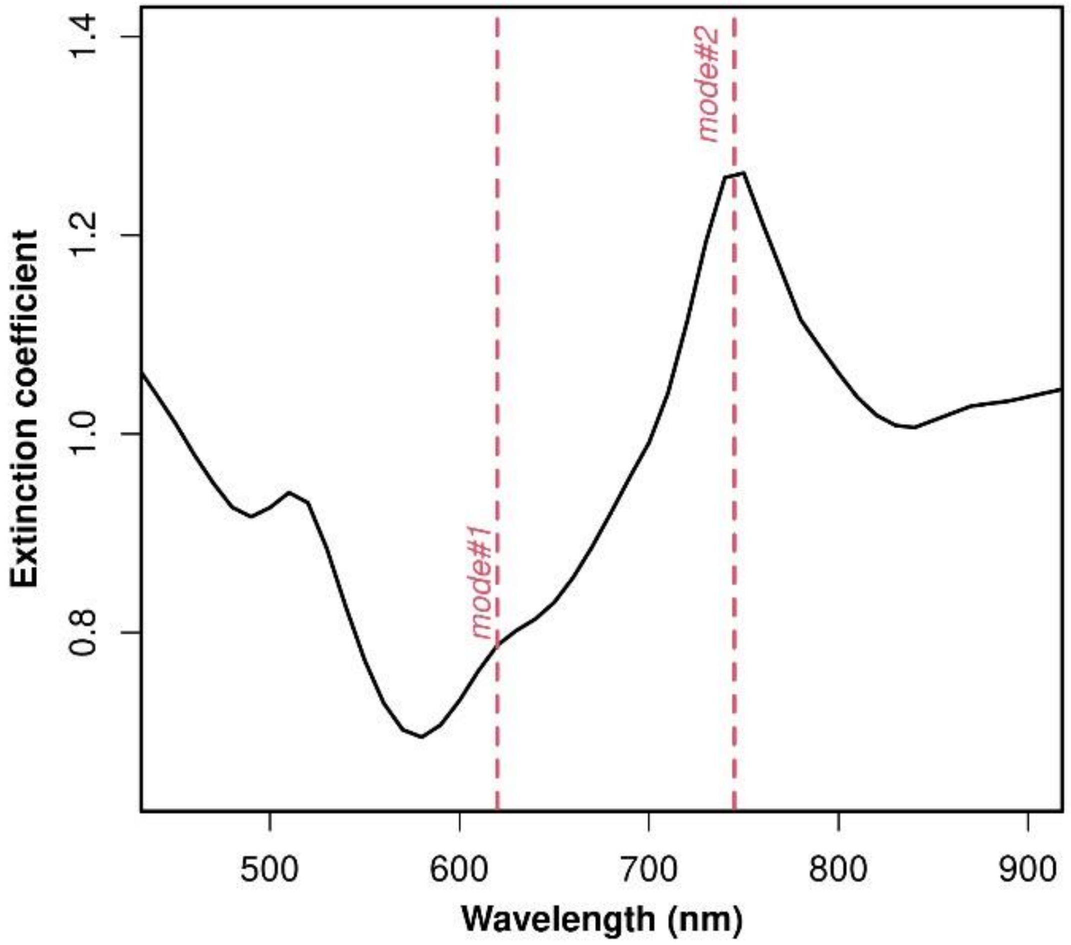

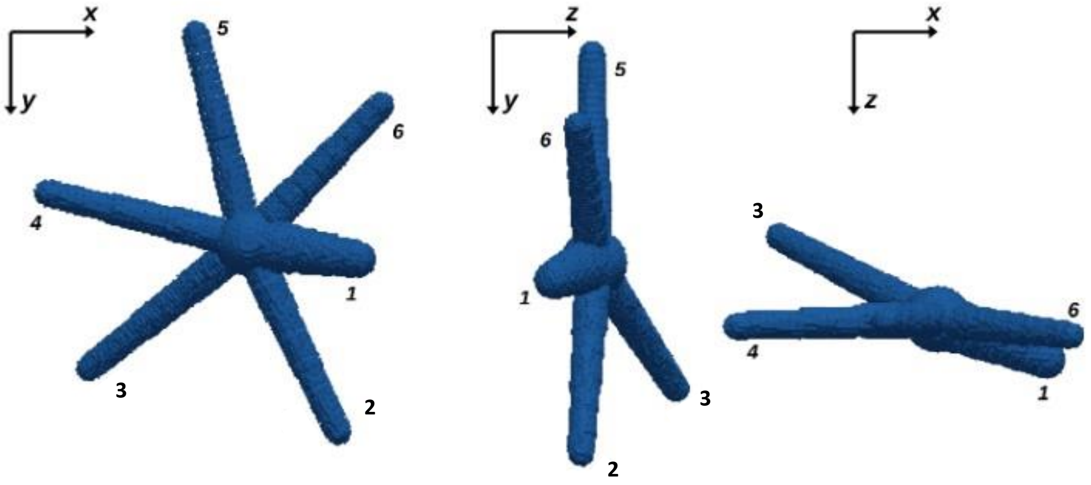

Figure S25. Top: DDA simulated extinction spectra for the measured nanostar 3D morphology. Bottom DDA simulated nanoparticle shape presented by three orientations, axis representation and leg numbering as used in Figure 1.

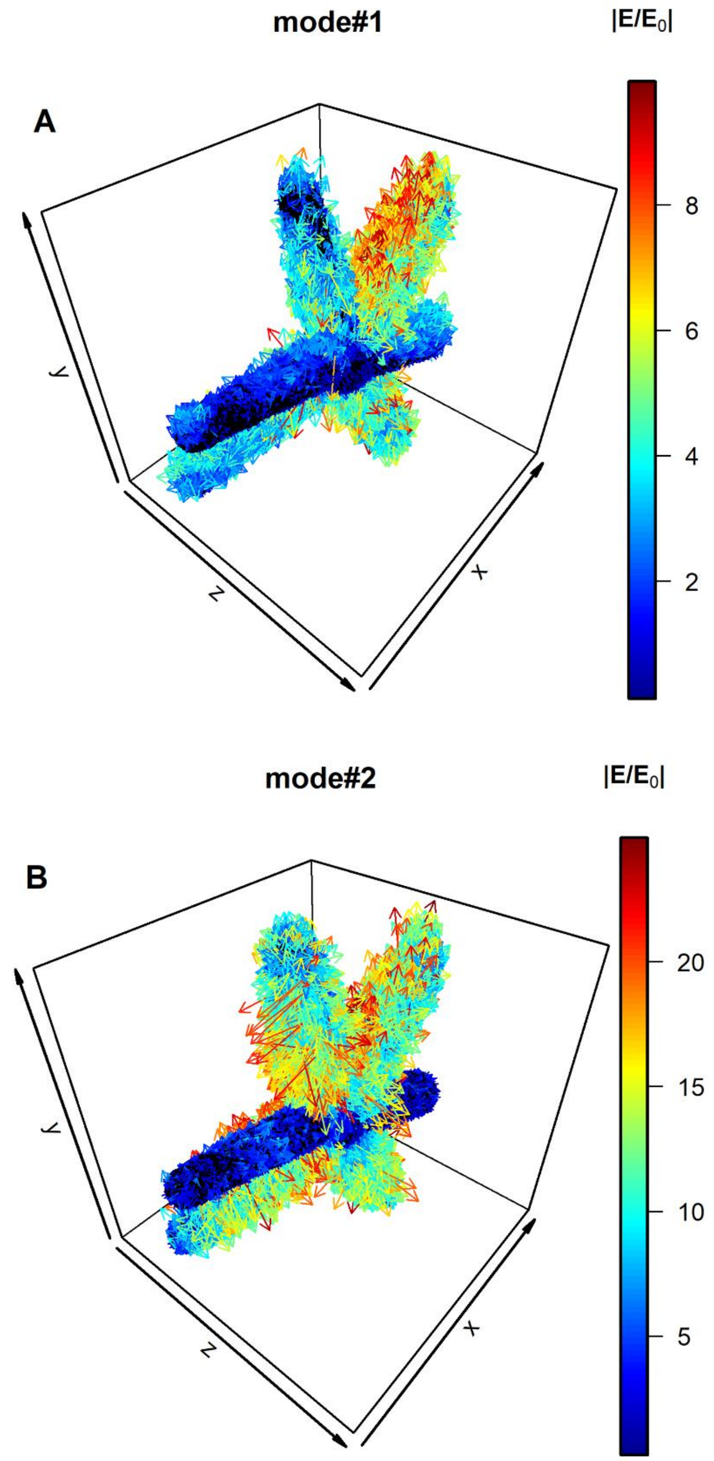

Figure S26. Polarization vectors representation in terms of the real part of the electric field at the dipole positions for mode#1 (A) and mode#2 (B). Colors represent the electric field enhancement.

References

1. C. B. Carter, D. B. Williams, Eds., *Transmission Electron Microscopy* (Springer International Publishing, Cham, 2016).

2. J. M. Zuo; J. C. H. Spence; *Advanced Transmission Electron Microscopy* (Springer New York, New York, NY, 2017).

3. E. J. Kirkland, *Advanced Computing in Electron Microscopy* (Springer International Publishing, Cham, 2020).

4. Vincent, R.; Midgley P. A. Double Conical Beam-Rocking System for Measurement of Integrated Electron Diffraction Intensities. Ultramicroscopy 1994, 53, 271–282.

5. Rauch, E. F.; Portillo, J.; Nicolopoulos, S.; Bultreys, D.; Rouvimov, S.; Moeck, P. Automated Nanocrystal Orientation and Phase Mapping in the Transmission Electron Microscope on the Basis of Precession Electron Diffraction. Z. Kristallogr. 2010, 225 (2–3), 103–109.

6. Midgley, P. A.; Eggeman, A. S. Precession Electron Diffraction – A Topical Review. IUCrJ 2015, 2, 126–136.

7. P. Oleynikov, S. Hovmöller, X. D. Zou, Precession electron diffraction: Observed and calculated intensities. *Ultramicroscopy* **107**, 523–533 (2007).

8. Own, C. S.; Marks, L. D.; Sinkler, W. Precession electron diffraction 1: multislice simulation. *Acta Crystallogr. A Found. Crystallogr.* **2006**, 62, 434–443.

9. Cautaerts, N.; Crout, P.; Ånes, H. W.; Prestat, E.; Jeong, J.; Dehm, G.; Liebscher, C. H. Free, Flexible and Fast: Orientation Mapping Using the Multi-Core and GPU-Accelerated Template Matching Capabilities in the Python-Based Open Source 4D-STEM Analysis Toolbox Pyxem. Ultramicroscopy 2022, 237, 113517.

10. Ophus, C.; Zeltmann, S. E.; Bruefach, A.; Rakowski, A.; Savitzky, B. H.; Minor, A. M.; Scott, M. C. Automated Crystal Orientation Mapping in py4DSTEM Using Sparse Correlation Matching. Microsc Microanal 2022, 28 (2), 390–403.

11. Corrêa, L. M.; Ortega, E.; Ponce, A.; Cotta, M. A.; Ugarte D. High Precision Orientation Mapping from 4D-STEM Precession Electron Diffraction Data Through Quantitative Analysis of Diffracted Intensities. Ultramicroscopy 2024, 259, 113927.

12. Palatinus, L.; Brázda, P.; Jelínek, M.; Hrdá, J.; Steciuk, G.; Klementová, M. Specifics of the Data Processing of Precession Electron Diffraction Tomography Data and Their Implementation in the Program PETS2.0. Acta Crystallogr. B Struct. Sci. Cryst. Eng. Mater. 2019, 75 (4), 512–522.

13. Eggeman, A. S.; Krakow, R.; Midgley, P. A. Scanning Precession Electron Tomography for Three-Dimensional Nanoscale Orientation Imaging and Crystallographic Analysis. Nat. Commun. 2015, 6, 7267.

14. M. De Graef, *Introduction to Conventional Transmission Electron Microscopy* (Cambridge University Press, ed. 1, 2003)

15. Gallinet, B.; Butet, J.; Martin, O. J. F. Numerical Methods for Nanophotonics: Standard Problems and Future Challenges. Laser Photonics Rev. 2015, 9, 577–603.

16. Yang, C. Y. Crystallography of Decahedral and Icosahedral Particles. J. Cryst. Growth 1979, 47, 274–282.

17. Francisco de la Peña; Eric Prestat; Vidar Tonaas Fauske; Pierre Burdet; Jonas Lähnemann; Petras Jokubauskas; Tom Furnival; Carter Francis; Magnus Nord; Tomas Ostasevicius; Katherine E. MacArthur; Duncan N. Johnstone; Mike Sarahan; Joshua Taillon; Thomas Aarholt; pquinn-dls; Vadim Migunov; Alberto Eljarrat; Jan Caron; T. Nemoto; Timothy Poon; Stefano Mazzucco; actions-user; Nicolas Tappy; Niels

Cautaerts; Suhas Somnath; Tom Slater; Michael Walls; pietsjoh; Hugh Ramsden. Hyperspy/Hyperspy: V2.0.1, 2024. https://doi.org/10.5281/ZENODO.10709941.

18. Seto, Y.; Ohtsuka, M. ReciPro: Free and Open-Source Multipurpose Crystallographic Software Integrating a Crystal Model Database and Viewer, Diffraction and Microscopy Simulators, and Diffraction Data Analysis Tools. *J. Appl. Crystallogr.* **2022**, 55, 397–410.

19. De Graef, M. & McHenry, M. E. Structure of Materials: *An Introduction to Crystallography, Diffraction and Symmetry*; Cambridge University Press, Cambridge, 2012.
